# Treatment of Substandard Rocket Fuel 1,1-Dimethylhydrazine via Its Methylene Derivative into Heterocycles Based on Pyrrolo-[3,4c]Quinolines, Cyclododeca[b]piran and Pyrrole

**DOI:** 10.3390/ijms241713076

**Published:** 2023-08-22

**Authors:** Elizaveta Ivanova, Maxim Maryasov, Vera Andreeva, Margarita Osipova, Tatyana Vasilieva, Alexey Eremkin, Olga Lodochnikova, Denis Grishaev, Oleg E. Nasakin

**Affiliations:** 1 Organic and Pharmaceutical Chemistry Department, ssUlyanov Chuvash State University, Moskovsky Prospect, 15, 428015 Cheboksary, Russia; lizachimic@mail.ru (E.I.); marsikprovisor@mail.ru (M.M.); ver.92.92@mail.ru (V.A.); margolev1966@icloud.com (M.O.); tava52@mail.ru (T.V.); eremkin80@mail.ru (A.E.); 2 Organic and Pharmaceutical Chemistry Department, Arbuzov Institute of Organic and Physical Chemistry, FRC Kazan Scientific Center of RAS, Arbuzov Streed, 8, 420088 Kazan, Russia; lod_olga@mail.ru; 3 Organic and Pharmaceutical Chemistry Department, Scientific and Educational Center Pharmacy, Kazan Federal University, Paris Commune Street, 9, 296100 Kazan, Russia; dionis.grishaev@yandex.ru

**Keywords:** unsymmetrical dimethylhydrazine, methylene dimethylhydrazone, tetracyanoethylene, tetracyanoketones

## Abstract

1,1-Dimethylhydrazine (Heptil, rocket fuel (UDMH)) is characterized by extremely high toxicity, teratogenicity and the ability to constantly absorb water from the atmosphere, losing its energy characteristics. In this regard, as well as due to the alternative fuel (“Angara”) transition, there is a need for UDMH utilization in huge amounts. A more benign approach involves its immediate reaction with a formalin solution to form 1,1–dimethyl-2-methylene hydrazone (MDH), which is significantly less toxic by an order of magnitude. MDH can then be polymerized under acidic conditions, and the resulting product can be burned, yielding a substantial amount of nitrogen oxides. We propose an alternative to incineration by involving MDH in organic synthesis. We studied the reactions of MDH and its analog N,N-dimethyl-2-(methylenamino)ethane-1-amine (MDEA) with available CH-acids: tetracyanoethylated ketones (TCEKs) based on cyclohexanone, 4-propylcyclohexanone, 2-methylcyclohexanone, cyclododecanone and tetracyanoethane. The structures synthesized were confirmed by IR, ^1^H, ^13^C NMR and mass spectroscopy methods. MDH-based adducts were also identified by X-ray structural analysis. TCEKs and MDH, as well as TCEK based on cyclohexanone and MDEA, form bi- and tricyclic structures: pyrrolo [3,4c]-quinolines (using TCEKs based on cyclohexanone and 4-propylcyclohexanone), epiminomethanoquinoline-3,4-dicarbonitrile (using TCEK based on 2-methylcyclohexanone) and cyclododec[b]pyran-3,4-dicarbonitrile (using TCEK based on cyclododecanone). MDH and TCNEH_2_ formed a pyrrole derivative. Thus, we synthesized the structures that are of interest for molecular design and pharmaceutical chemistry.

## 1. Introduction

Heptil (unsymmetric dimethylhydrazine, UDMH) is actively used worldwide as a fuel for rocket engines. At the same time, it constantly loses its properties due to uncontrolled absorption of moisture that cannot be separated by rectification. UDMH is an extremely toxic compound (Hazard class 1), which complicates its transportation for treatment. During disposal by incineration, some oxidation products that are much more toxic than the original substance are produced. The safest way to neutralize it is its instantaneous interaction with a formalin solution to form a less toxic 1,1-dimethyl-2-methylene hydrazone (MDH) [1,2], that is then polymerized in an acidic medium, with the resulting product incinerated producing a huge amount of nitrogen oxides. This study aims to identify the synthetic abilities and practical applications of MDH (instead of incineration). We would like to draw the chemical community’s attention to this metastable compound, which is promising for use in molecular design, as well as in pharmaceutical and medicinal chemistry. We have studied the reactions of MDH with polycyano-containing CH-acids. As the latter, available tetracyanoethylated ketones (TCEKs) were used, which are the most stable and prone to cascade transformations, and they have a high synthetic potential (readily available CH-acids, pKa 2.8–3.6 [3]). The main properties of MDH [1] suggested catalytic intramolecular cyclizations with the formation of a fragment—pyrrolidin-2-one.

**Figure 1:**
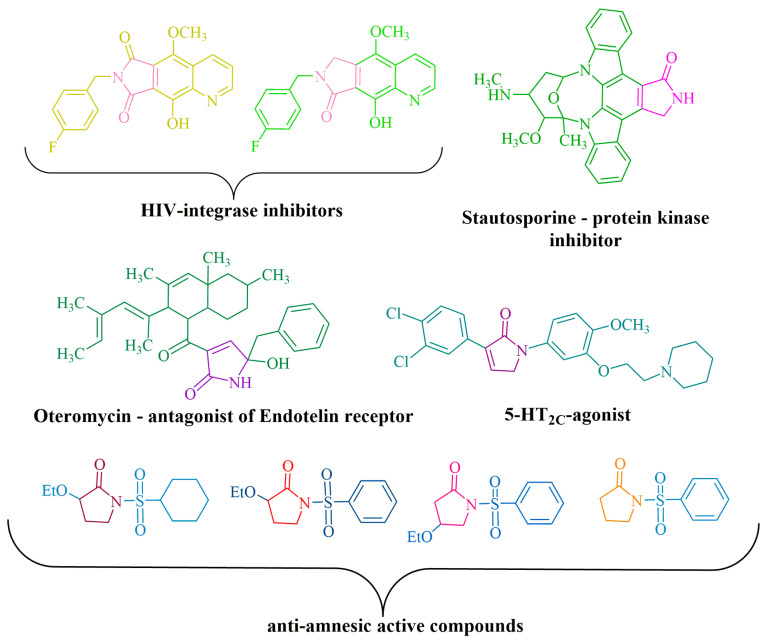
Biologically active compounds containing the moiety of pyrrolidin-2-one.

This fragment represents a group of inhibitors belonging to various enzyme classes, including HIV-1 integrase [4] (an enzyme responsible for catalyzing the integration of HIV-1 viral DNA into the host cell chromosome), tyrosine kinase [5] (an enzyme that facilitates the transfer of the phosphate group from ATP to tyrosine residue), and telomerase [6] (an enzyme that adds a specific sequence to the end of the DNA chain and stabilizes the chromosomes). Additionally, it serves as a structural component in agonists, namely chemical compounds which elicit biological responses upon interaction with receptors. Examples of such agonists include serotonin [7], chemokine [8] (a peptide that regulates leukocyte movement and their migration from the bloodstream into tissues), and endothelin [6] (a potent vasoconstrictor receptor composed of 21 amino acids). Furthermore, the compound pyrrolidin-2-one is utilized as an active ingredient in drugs employed for the treatment of disorders associated with neurological function, memory and mental fatigue [9] (Figure 1).

**Figure 2:**
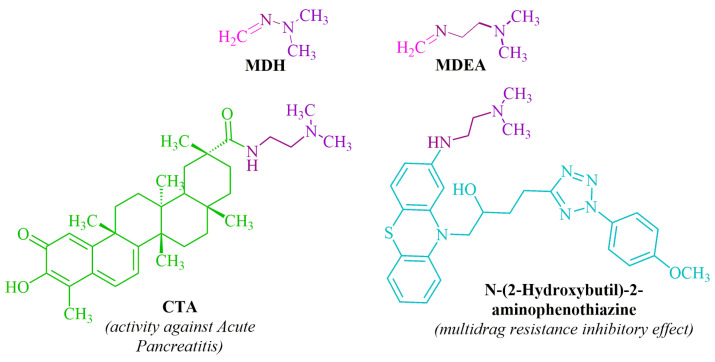
Structural analogs **MDH**, **MDEA** and **DMDEA**-containing biologically active compounds.

The methylene derivative of N,N-dimethyl-2-(methylenamino)ethane-1-amine (MDEA), known as N,N-dimethylethane-1,2-diamine (DMEDA), possesses spaced reaction centers and undergoes reactions with TCEKs similar to MDH. Furthermore, this fragment is integral to medications employed in the treatment of acute pancreatitis [10] and the mitigation of drug resistance in cancer cells [11] (Figure 2).

**Figure 3:**
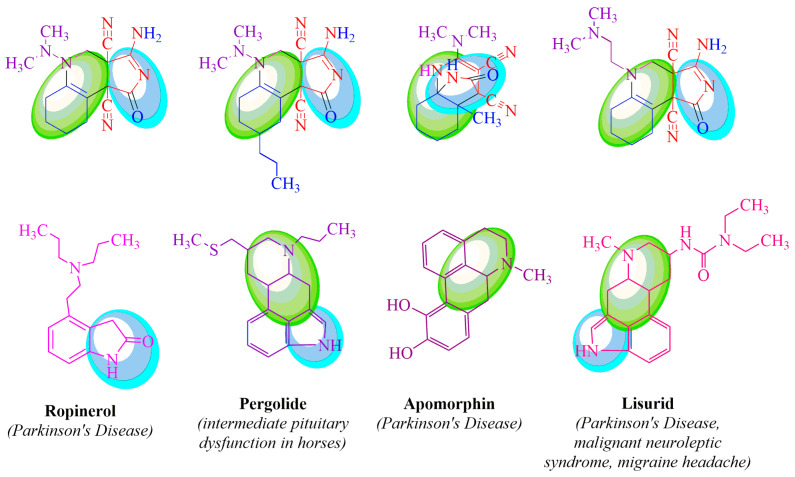
Structural similarities in TCEKs derivatives and well-known drugs.

The compounds synthesized in this study, derived from TCEKs, MDH, and MDEA, exhibit structural similarities to well-known drugs due to the presence of hydroquinoline moieties (highlighted by the green circle in Figure 3) and pyrrole moieties (highlighted by the blue circle in Figure 3).

As a result, pyrrolo[3,4*c*]-quinolines (obtained through MDH and TCEK reactions using cyclohexanone and 4-propylcyclohexanone) and epiminomethanoquinoline-3,4-dicarbonitrile (synthesized via MDH and TCEK reactions using 2-methylcyclohexanone) exhibit structural similarities to compounds utilized in the treatment of neurodegenerative diseases (Figure 3) (Ropinirole [12], Pergolide [13,14], Lisuride [15,16,17] and Apofomin [18]).

**Figure 4:**
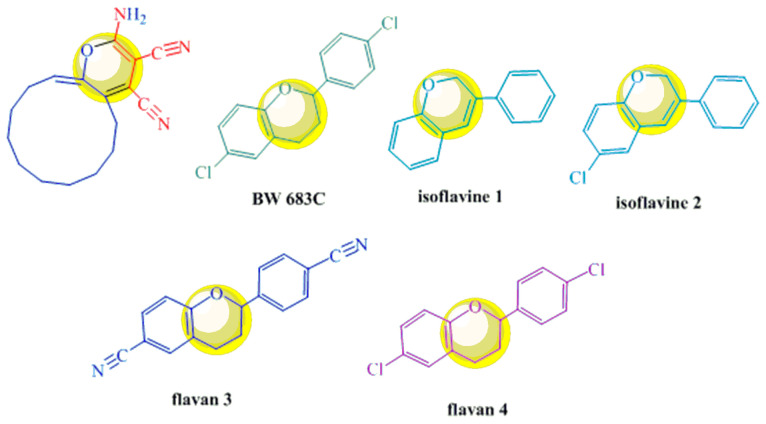
Structural similarities in cyclododecanone derivatives and flavanoids exhibiting antienteroviral activity.

Cyclododeca[b]pyran-3,4-dicarbonitrile (synthesized through the use of 2-methyl-5-decylhexane-2,5-dione and trichloroethyl ketone based on cyclododecanone) is expected to find application as an antiviral agent, owing to the presence of the pyran ring, which is a common structural motif in biologically active antiviral compounds [19] (Figure 4).

## 2. Results

### 2.1. Results of the Syntheses

#### 2.1.1. Structures Based on **MDH** and **MDEA** with **TCEKs 1a,b,c** and **2**

- pyrrolo[3,4*c*]-quinolines **9a,b** (**TCEKs 1a,b** with **MDH**) (see Figure 6, Section 3 Discussion);
- **9′** (TCEK **1a** with **MDEA**) (see Figure 6, Section 3).
- 8a,4-(epiminomethano)quinoline-3,4-dicarbonitrile **11c** (**TCEK 1c** with **MDH**) (see Figure 7, Section 3);
- cyclodode[*b*]pyran-3,4-dicarbonitrile **8** (**TCEK 2** with **MDH**) (see Figure 9, Section 3).

#### 2.1.2. Structures Based on **MDH** with **TCNEH_2_**

- pyrrole derivative **12** (see Figure 14, Section 3);

The structures obtained were determined by IR, ^1^H, ^13^C NMR and mass spectroscopy (the description of some chemical shifts see in Section 3). The data are shown in the table below (Table 1) (see Figures S1–S18 in Supplementary Materials): 

#### 2.1.3. Elemental Analysis of the Structures Obtained

- **3-Amino-5-(dimethylamino)-1-oxo-4,5,6,7,8,9-hexahydro-1*H*-pyrrolo[3,4-*c*]quinoline-3a,9b-dicarbonitrile (9a)**. Found: C 60.28; H 6.31; N 28.05. C_15_H_18_N_6_O. Calculated %: C 60.39; H 6.08; N 28.17.
- **3-Amino-5-(dimethylamino)-1-oxo-8-(propyl)-4,5,6,7,8,9-hexahydro-1*H*-pyrrolo[3,4-*c*]quinoline-3a,9b- dicarbonitrile (9b)**. Found: C 63.48; H 7.13; N 24.65. C_18_H_24_N_6_O. Calculated %: C 63.51; H 7.11; N 24.69.
- **3-Amino-5-(2-(dimethylamino)ethyl-1-oxo-4,5,6,7,8,9-hexahydro-1*H*-pyrrolo[3,4-*c*]quinoline-3a,9b-dicarbonitrile (9′)** Found: C 62.53 H 6.75 N 25.80. C_17_H_22_N_6_O. Calculated %: C 62.56; H 6.79; N 25.75.
- **2-(Dimethylamino)-4a-methyl-10-oxo-5,6,7,8-tetrahydro-1*H*-8a,4-(epimino)quinoline-3,4(4a*H*)-dicarbonitrile (12c)**. Found: C 63.08; H 6.69; N 24.55. C_15_H_19_N_5_O. Calculated %: C 63.14; H 6.71; N 24.54.
- **(E)2-Amino-6,7,8,9,10,11,12,13-octahydro-5*H*-cyclododeca [*b*]pyran-3,4-dicarbonitrile (8)**. Found: C 72.03; H 7.42; N 14.80. C_17_H_21_N_3_O. Calculated %: C 72.06; H 7.47; N 14.83.
- **5-Amino-1-(dimethylamino)-1,2-dihydro-3*H*-pyrrole-3,3,4-tricarbonitrile (12)**. Found, %: C 53.61; H 4.72; N 41.67. C_9_H_10_N_6_. Calculated %: C 53.46; H 4.98; N 41.56.

#### 2.1.4. Crystal Data on the Structures **9a,b, 12c** and **8** (See the Descriptions in Section 3, Figures 8–10 and 12)

- **Crystal Data** for **9a**: C_15_H_18_N_6_O (*M* =298.35 g/mol): triclinic, space group P-1 (no. 2), *a* = 13.9756(12) Å, *b* = 15.0773(15) Å, *c* = 18.0923(13) Å, *α* = 112.010(8)°, *β* = 93.942(7)°, *γ* = 112.380(9)°, *V* = 3167.3(5) Å^3^, *Z* = 8, *T* = 104(7) K, μ(Cu Kα) = 0.683 mm^−1^, *Dcalc* = 1.251 g/cm^3^, 34121 reflections measured (5.436° ≤ 2Θ ≤ 156.656°), 12415 unique (*R*_int_ = 0.1673, R_sigma_ = 0.1628) which were used in all calculations. The final *R*_1_ was 0.0845 (I > 2σ(I)) and *wR*_2_ was 0.2282 (all data). CCDC number 2152262.
- **Crystal Data** for **9b**: C_22_H_32_N_6_O_3_ (*M* =428.53 g/mol): triclinic, space group P-1 (no. 2), *a* = 9.42370(10) Å, *b* = 11.97650(10) Å, *c* = 11.99720(10) Å, *α* = 111.2460(10)°, *β* = 111.1820(10)°, *γ* = 93.9690(10)°, *V* = 1145.11(2) Å^3^, *Z* = 2, *T* = 102.2(9) K, μ(Cu Kα) = 0.690 mm^−1^, *Dcalc* = 1.243 g/cm^3^, 33743 reflections measured (8.14° ≤ 2Θ ≤ 153.026°), 4548 unique (*R*_int_ = 0.0349, R_sigma_ = 0.0172) which were used in all calculations. The final *R*_1_ was 0.0422 (I > 2σ(I)) and *wR*_2_ was 0.1101 (all data). CCDC number 2152263.
- **Crystal Data** for **12c**: C_15_H_19_N_5_O (*M* =285.35 g/mol): triclinic, space group P-1 (no. 2), *a* = 7.5867(2) Å, *b* = 9.1339(3) Å, *c* = 10.8470(4) Å, *α* = 76.621(3)°, *β* = 82.305(3)°, *γ* = 81.452(3)°, *V*= 719.26(4) Å^3^, *Z* = 2, *T* = 100(1) K, μ(Cu Kα) = 0.703 mm^−1^, *Dcalc* = 1.318 g/cm^3^, 17589 reflections measured (8.424° ≤ 2Θ ≤ 152.906°), 2865 unique (*R*_int_ = 0.0345, R_sigma_ = 0.0220) which were used in all calculations. The final *R*_1_ was 0.0399 (I > 2σ(I)) and *wR*_2_ was 0.0993 (all data). CCDC number 2152264.
- **Crystal Data** for **8**: for C_17_H_21_N_3_O (*M* =283.37 g/mol): triclinic, space group P-1 (no. 2), *a* = 8.5574(5) Å, *b* = 9.7008(6) Å, *c* = 9.7930(4) Å, *α* = 102.032(4)°, *β* = 103.106(4)°, *γ* = 101.505(5)°, *V* = 747.97(7) Å^3^, *Z* = 2, *T* = 99.9(10) K, μ(Cu Kα) = 0.632 mm^−1^, *Dcalc* = 1.258 g/cm^3^, 7141 reflections measured (9.618° ≤ 2Θ ≤ 153.2°), 2981 unique (*R*_int_ = 0.0614, R_sigma_ = 0.0595) which were used in all calculations. The final *R*_1_ was 0.0821 (I > 2σ(I)) and *wR*_2_ was 0.2326 (all data). CCDC number 2152265.

See crystal data and structure refinement for **9a**, **9b**, **12c** and **8** in the table below (Table 2):

## 3. Discussion

**Figure 5:**
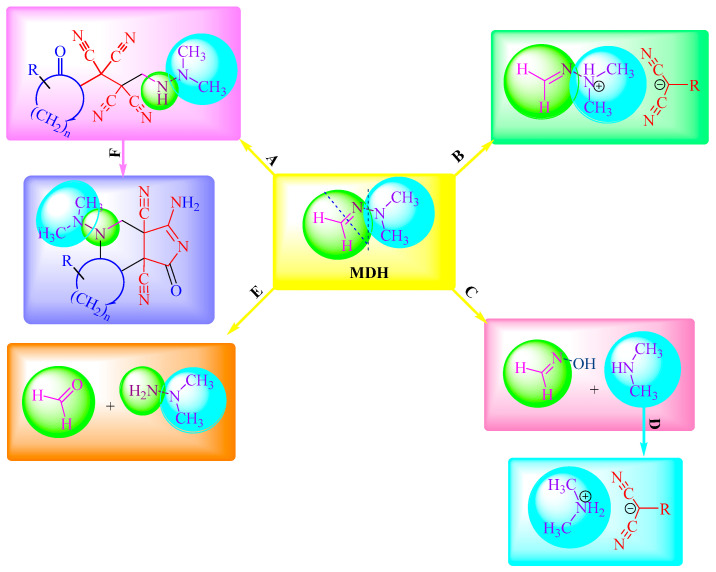
**MDH** reaction centers.

It appears that **MDH** easily reacts with **TCEKs** via all its structural fragments: Methylene group (A) ensures cyclization to pyrrolidin-2-one (F) and the dimethylamine fragment (B). We found out that MDH decomposed during its interactions, forming original compounds (E) and degraded further into dimethylamine and formaldoxime (C). In turn, the intermediate in the C-direction was able to produce an appropriative salt (direction D, Figure 5).

All of the directions above are implemented in the examples below.

All of the syntheses were carried out in a basic medium of ethyl acetate at room temperature. Chemical **TCEK** transformations [20] in an alkaline medium are described in our earlier publication [21]. However, spiro compounds obtained there [21] differed from the structures presented herein. The structures obtained by morpholine catalysis are also different [22].

**Figure 6:**
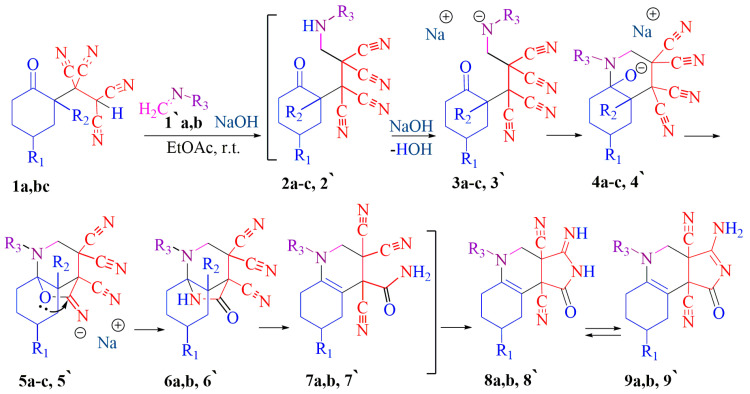
Reactions of cyclohexanone **1a**, 4-propylcyclohexanone **1b**, and 2-methylcyclohexanone **1c** derivatives with **MDH 1′a** and cyclohexanone **1a** with **MDEA 1b’**.

Thus, 1-(2-oxocyclohexyl)ethane-1,1,2,2-tetracarbonitrile **1a**, 1-(2-oxo-5-propylcyclohexyl)ethane-1,1,2,2-tetracarbonitrile **1b** with **MDH (1′a)** (intermediates **2a,b–8a,b**) and cyclohexanone derivative **1a** with **MDEA (1′b)** (intermediates **2′–8′**) interacted according to the same scheme (Figure 6). The derivative of 2-methylcyclohexanone **1c** with **MDH** (**1′a**) undergoes chemical transformations identical to **TCEKs 1a** and **1b** (intermediates **2c–5c**) until the moment of rearrangement.

**Figure 7:**
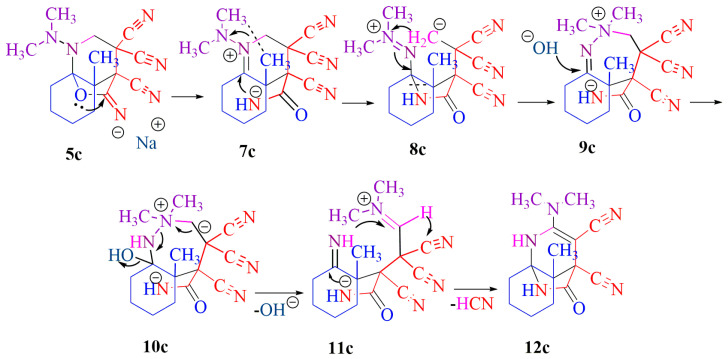
Further chemical transformations of TCEK **1c**.

Presumably, the first stage of the interaction is the same for three **TCEKs 1a–c** and is a Michael-type attachment of the CH-acid center **1a–c** to **1′a,b**. The resulting Michael-type adducts **2a–c**, **2′** are likely to undergo intermolecular cyclization transformations catalyzed by alkali. Zwitterions **3a–c**, **3′** via intermediates **4a–c**, **4′** transform into heterocycles **5a–c**, **5′**. The rearrangement of the 2-methylcyclohexanone derivative **5c** is carried out according to another scheme (Figure 7). Tricycles **6a,b, 6′** open into amides **7a,b, 7′**. Thorpe–Ziegler type cyclization [23] in γ-lactam leads to pyrrolo[3,4-*c*]quinolines **MDH**-based **9a** (*m*/*z* 299.1620) (see Figure S3 in Supplementary Materials), **9b** (*m*/*z* 341.2090) (see Figure S6 in Supplementary Materials) and **MDEA**-based **9′** (*m*/*z*: 326.0000) (see Figure S9 in Supplementary Materials) (Figure 6).

The substituents in the structures mentioned above are presented in the table below (Table 3).

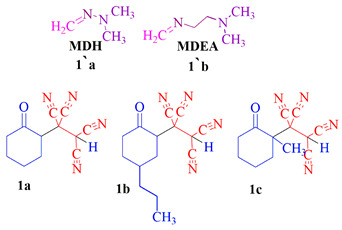



The absence of a proton in the α-position of **TCEK 1c** causes a different direction of the chemical process. Therefore, after the Thorpe–Ziegler type cyclization (intermediate **5c**, Figure 6), we assume an electron density redistribution during the rearrangement. This may lead to the opening of a six-membered cycle **7c** and to the cyclization of diazene **8c** into 1,2-diazepine **9c** (Figure 7). The activated N=N+ bond promotes cyclization into 1,2-diazepine, according to the publication [24]. The transition of N-aminopyridine derivatives to a diazepine intermediate is also described in the article [25]. The alkaline medium is likely to promote the N-N bond cleavage of the seven-membered cycle **9c** with subsequent cyclization into α–(*N,N*-dimethylamino)tetrahydropyridine **12c** by analogy with the publication [26]. Prussic acid elimination during the cyclization of intermediate **11c** was confirmed by a qualitative reaction to Prussian blue (see Section 3).

Previously, we proposed the formation of the desired product **12c** through the decomposition of **MDH** into prussic acid and dimethylamine (**DMA**), which was subsequently added to the terminal carbonitrile of **TCEK 1c**. The hypothesis of **MDH** decomposition into DMA was tested using gas chromatography (refer to Section 4). The gas-chromatographic analysis results showed no presence of **DMA** in the reaction mixture. The retention time of **DMA** synthesized using a standard procedure [27] was observed at 120 s. The retention times of the gases released during the reaction of **TCEK 1c** and **MDH** were 30 s and 7 min. Therefore, the formation of compound **12c** according to the proposed schemes (Figure 6 and Figure 7) is more probable.

**Figure 8:**
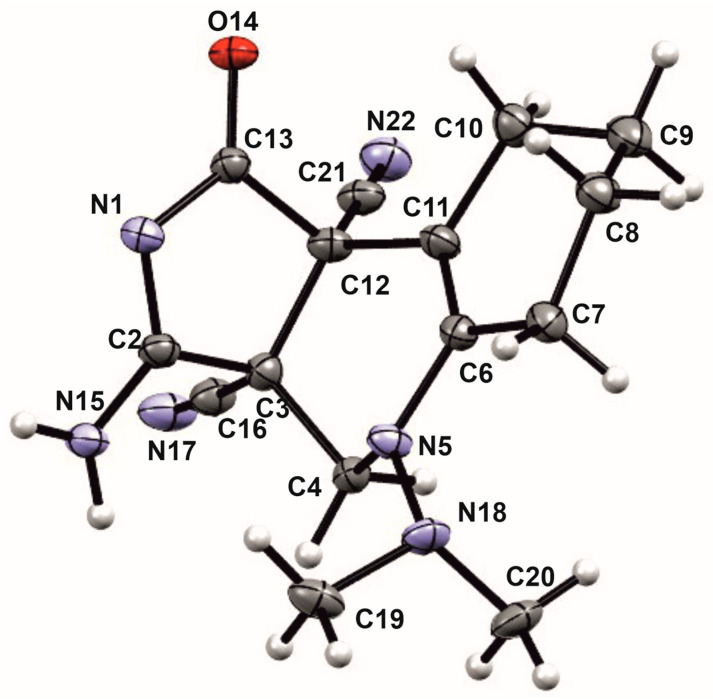
The crystal structure of **9a**.

**Figure 9:**
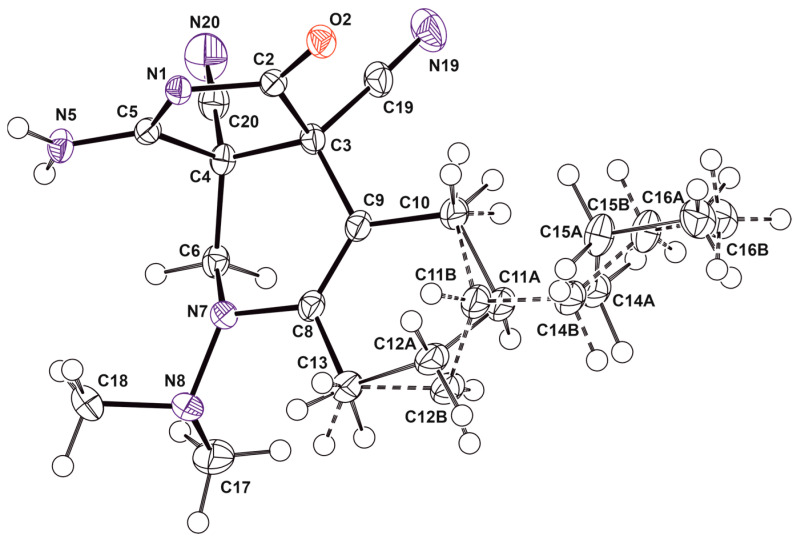
The crystal structure of **9b**.

**Figure 10:**
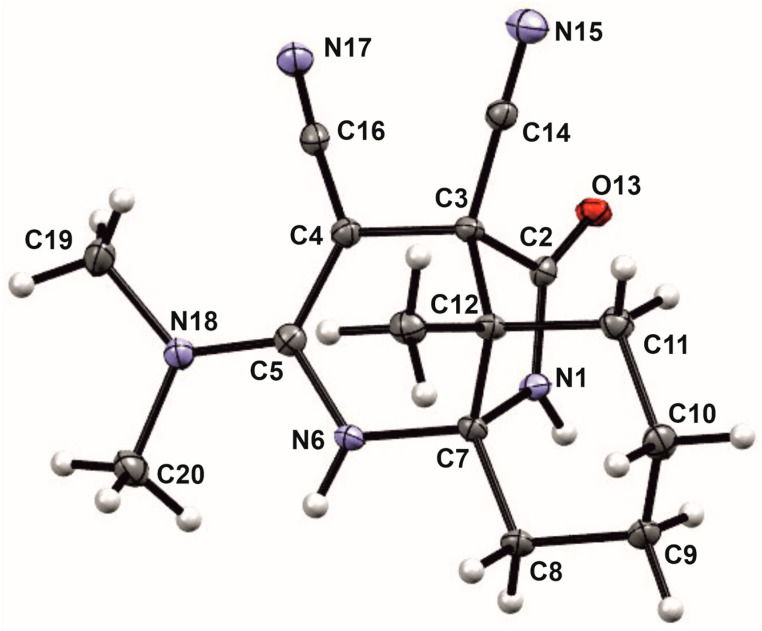
The crystal structure of **11c**.

The unusual structures of the resulting compounds were determined using proton nuclear magnetic resonance (^1^H NMR) (see Figures S1, S4, S7, S10, S13 and S16 in Supplementary Materials), carbon nuclear magnetic resonance (^13^C NMR) (see Figures S2, S5, S8, S11, S14 and S17 in Supplementary Materials), and X-ray diffraction analysis (Table 1 and Table 2, Section 2. Results). Descriptions of the crystal structures of the resulting compounds **9a**, **9b** and **11c** are provided below (Figure 8, Figure 9 and Figure 10).

Compound **9a** is represented in the crystal by four independent molecules with the same geometry shown in Figure 8.

In crystal **9b**, disorder was found in the fragment containing a propyl substituent, a chiral center at C11 and a methylene group at C12. This indicates the presence in the crystal of two diastereomers differing in the configuration of the C11 atom (Figure 9).

**Figure 11:**
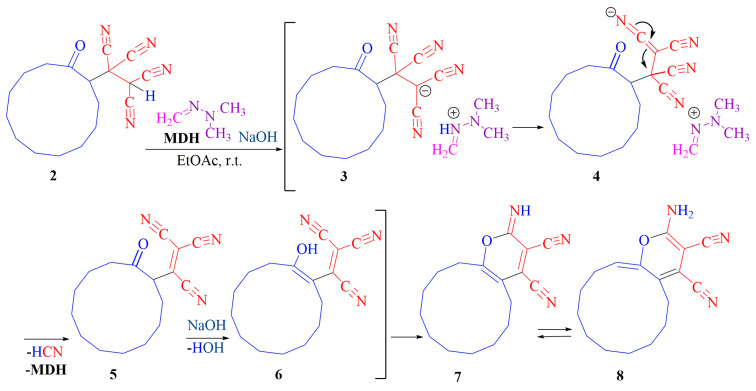
Interaction between TCEK **2** and **MDH**.

The macrocyclic cyclododecanone derivative **2** is stable and does not react with **MDH**. However, the latter may potentially deprotonate **TCEK 2**. Subsequently, salt **3** is expected to undergo transformation into tricyanovinyl intermediate **5**, following a similar process described in publication [28]. Intermediate **5** then undergoes tautomerization to form enolate **6** under basic conditions, which facilitates cyclization to yield pyran derivative **8** (*m*/*z* 283.1700) (see Figure S15 in Supplementary Materials) (Figure 11). The presence of prussic acid was confirmed through a qualitative reaction with Prussian blue (refer to Section 4).

To evaluate the efficacy of **MDH** as a catalyst for **TCEK 2**-related reactions, we performed a control synthesis of compound **8** without the inclusion of **MDH** in the reaction mixture (refer to Section 4). The formation of the desired product **8** was observed within 24 h, whereas the **MDH**-catalyzed reaction proceeded within 30 min.

**Figure 12:**
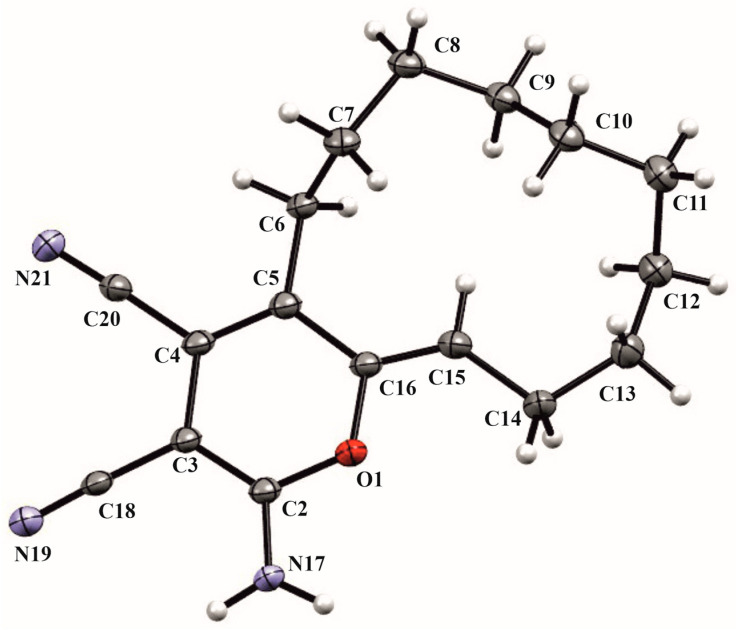
The crystal structure of **8**.

The structure of the resulting compound **8** is shown below (Figure 12).

**Figure 13:**
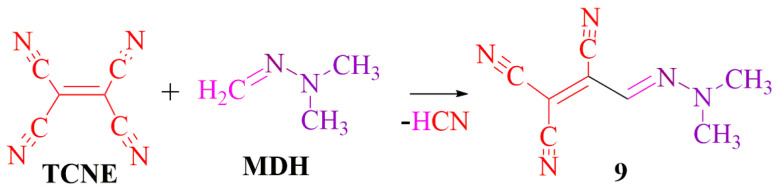
Interaction between **TCNE** and **MDH**.

**DMH** reacts with the tetracyanoethylene (**TCNE**) released at the hydrogen atom of the methylene group. In our earlier publication [29], we reported that the low-yield tricyanohydrazine derivatives **9** were obtained, known as potential antimicrobial dyes and photosensitizers (Figure 13).

**Figure 14:**
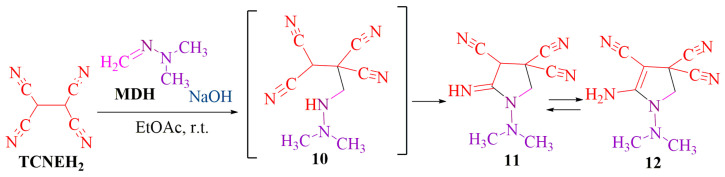
Interaction between **TCNEH_2_** and **MDH**.

The **MDH**-reaction at the CH-acidic center enables the incorporation of **MDH** in the synthesis with tetracyanoethane (**TCNEH_2_**). In contrast to our previous publication [29], which described the addition of **MDH** to tetracyanoethylene (**TCNE**) through methylene-active hydrogen, the reaction of **MDH** with **TCNEH_2_** leads to the formation of cyclic pyrrole derivative **12** (*m*/*z* 203.1047) (see Figure S18 in Supplementary Materials), as depicted in the proposed chemical transformation scheme presented below (Figure 14).

As mentioned above *(*Section 1), the resulting products **9a,b**, **9′**, **12c**, **8** contain the moieties that are similar with the structures of well-known drugs used for the treatment of neurodegenerative and viral diseases. Therefore, the compounds obtained will be tested on biological activity in these areas.

## 4. Materials and Methods

The syntheses involving the release of hydrogen cyanide (prussic acid) should be conducted with the provision of a gas vent, an absorption flask and performed under a properly functioning hume hood. It is essential to use protective eyewear and latex gloves.

All reagents were procured from commercial vendors and employed without undergoing additional purification. Among them there is non-marketable rocket fuel based on unsymmetrical dimethylhydrazine (“Chemical Point ug”, Deisenhofen, Germany), formalin (“Across Organics”, Geel, Belgium), cyclohexanone (“Qingdao Honghao Chemical Co., Ltd.”, Shandong, China), 4-propyl cyclohexanone (“Santa Cruz Biotechnology”, Dallas, TX, USA), 2-methylcyclohexanone (“Across Organics”, Geel), cyclododecanone (“Hangzhou Keying Chem Co., Ltd.”, Hangzhou, China), ethyl acetate (“Across Organics”, Geel), tetracyanoethylene (“Across Organics”, Geel), iron sulfate (“Across Organics”, Geel), potassium hydroxide (“Across Organics”, Geel).

The progress of reactions and the purity of products were monitored via thin-layer chromatography (TLC) on Sorbfil plates (“Sorbfil”, Krasnodar, Russia). 

Visualization of spots was achieved under ultraviolet (UV) light, upon treatment with iodine vapor, or through heating. Melting and decomposition points were determined using the Optimelt MPA100 apparatus (“Optimelt”, Danbury, Connecticut). Infrared (IR) spectra were obtained using the FSM-1202 spectrometer equipped with Fourier transform technology, with samples dispersed in Nujol. Proton nuclear magnetic resonance (^1^H NMR) and carbon-13 nuclear magnetic resonance (^13^C NMR) spectra were acquired with the provision of the Carr−Purcell−Meiboom−Gill (CPMG) pulse sequence, employing DMSO-d_6_ solvent and utilizing the TMS internal standard. The measurements were conducted on a Bruker AVANCE 400 WB spectrometer (“Bruker”, Hanau, Germany) operating at the frequencies of 400.13 MHz for ^1^H and 100.61 MHz for ^13^C.

HRMS mass spectra of **9a**, **9b**, **12c** and **8** were acquired using the quadrupole time-of-flight (t, qTOF) AB Sciex Triple TOF 5600 mass spectrometer (AB SCIEX PTE. Ltd., Singapore) equipped with a turbo-ion spray source. The nebulizer gas used was nitrogen, and the ionization polarity was positive (+). The needle voltage was set at 5500 V. The spectra were recorded in the time-of-flight mass spectrometry (TOF MS) mode with a collision energy of 10 eV, declustering potential of 100 eV, and a resolution exceeding 30,000 full-width half-maximum. Sample solutions with an analyte concentration of 5 μmol/L were prepared by dissolving the test compounds in methanol (hypergrade for LC-MS, Merck).

An analytical reversed-phase HPLC was used for determination of uncalibrated purity of the compounds **7** and **28** and conducted using an Atlantis T3 C18 column (5µm, 150 × 4.6 mm); eluent A, 1.2% solution of triethylamine in water; eluent B CH_3_CN; and the gradient elution (0 min A:B = 85:15; 15 min A:B = 65:35; 21 min A:B = 65:35) flow rate was 1.0 mL/min. HPLC analysis was performed at 40 °C during 21 min at 311 nm.

Mass spectra of compound **9′** were acquired using the quadrupole gas-chromatography mass spectrometer (GCMS-QP2020 NX (“Shimadzu”, Duisburg, Germany)). Gas chromatograph was equipped with the column SH-I-5MS. Dimethyl polysiloxane was utilized as the stationary phase. A sample solution with an analyte concentration of 5 μmol/L was prepared by dissolving the test compound in isopropanol.

Data sets for single crystals **9a**, **9b**, **12c** and **8** were collected using the Rigaku Xta-Lab Synergy S instrument with a HyPix detector and a microfocus X-ray tube PhotonJet, utilizing Cu Kα radiation (1.54184 Å) at low temperature. The images were indexed and integrated using the data reduction package CrysAlisPro (“Rigaku Oxford Diffraction CrysAlisPro”, Oxford, UK). The data were corrected for systematic errors and absorption using the ABSPACK module: numerical absorption correction based on Gaussian integration over a polyhedron crystal model, and empirical absorption correction based on spherical harmonics according to the symmetry point group using equivalent reflections. The GRAL module was used for analyzing systematic absences and determining the space group. The structure was solved by direct methods using SHELXT [30] and refined by least squares with a full matrix on F2 using SHELXL [31]. Non-hydrogen atoms were refined anisotropically. Hydrogen atoms were placed in calculated positions and refined as riding atoms. Figures were generated using Mercury 4.1 software [32]. Crystals were obtained by the slow evaporation method. Crystal data and refinement parameters were summarized in Table 1 (Section 2).

Gases were identified using the Crystal 5000.1 laboratory gas chromatograph (CJSC SKB “Chromatek”, Yoshkar-Ola, Russia) with a thermo-electronic detector and the Chromatek-Analyst chromatographic data processing system under the following conditions: column temperature 100 °C; evaporator temperature 120 °C; detector temperature 390 °C; carrier gas flow rate (nitrogen) 20 cm^3^/min; hydrogen flow rate 14 cm3/min; and air flow rate 200 cm^3^/min.

**Qualitative reaction to prussic acid.** Firstly, 1–3 drops of a 40% solution of ferrum (II) sulfate were added to an absorption flask placed on a magnetic stirrer containing a dilute solution of potassium hydroxide while prussic acid gas was introduced through the gas vent. The solution was vigorously stirred and heated to boiling. Upon cooling of the reaction mixture, a 10% hydrochloric acid solution was added to achieve a slightly acidic reaction on universal paper. The appearance of blue staining and the formation of a blue precipitate confirmed the liberation of prussic acid during the reactions (Figure 7 and Figure 11, Section 3).

***N,N*-dimethyl-2-methylenehydrazone** (**MDH**) and *N,N*-dimethyl-2-(methylene-amino)ethan-1-amine (**MDEA**) were synthesized following a standard procedure [2] yielding 36% (3.89 g) and 38% (5.72 g), respectively.

To 0.15 mol of 38% formalin solution, 0.15 mol of **MDH** was added in small quantities, stirring the reaction mixture in an ice bath for an hour (since the reaction is exothermic). Then, NaOH was added in small quantities until two layers were formed in the reaction mixture. Then, the water layer was separated from the organic layer. The organic layer was distilled with the provision of a rectification column at a temperature of 64 °C in case of **MDH** and under reduced pressure in case of **MDEA**. The desired products **MDH** and **MDEA** were colorless liquids.

**Dimethylamine** (**DMA**) was obtained according to the method [27] with a yield of 85% (2.46 g).

Into a 50 mL-two-necked flask was placed N,N-dimethylamine hydrochloride (5.3 g, 65.0 mmol) suspended in DCM (15 mL), and finely powdered KOH (10.0 g, 180.0 mmol, 2.7 equiv.) was slowly added in portions to hold the temperature between −15 and −5 °C. After the mixture was stirred for 1 h, filtration led to a colorless solution of N,N-dimethylamine.

**1-(2-Oxocyclohexyl)ethane-1,1,2,2-tetracarbonitrile (1a), 1-(2-oxo-5-propylcyclohexyl)ethane-1,1,2,2-tetracarbonitrile (1b), 1-(1-methyl-2-oxohexyl-)ethane-1,1,2,2-tetracarbonitrile (1c), 1-(2-oxocyclododecyl)ethane-1,1,2,2-tetracarbonitrile (2)** were obtained according to the general procedure [20] with the yields of 74% (0.20 g), 75% (0.27 g), 66% (0.20 g) and 61% (0.24 g), respectively.

Then, 1.25 mmol tetracyanoethylene (**TCNE**) in 5 mL of dioxane was added to 1.22 mmol of ketone **1a**,**b**,**c**,**2** in 5 mL of dioxane along with a catalytic amount of hydrochloric acid (1 drop). The progress of the process was determined via a test for hydroquinone (HQ), that forms a blue p-complex when reacting with **TCNE**. After the blue color ceased to appear, the dioxane solution was maintained at the temperature range of 0–5 °C in the freezer for 10 min. After this period of time, cold distilled water was added to the frozen reaction mixture in a volume equal to the dioxane solution. The reaction mixture was stirred until the precipitation. The desired product was separated by filtration through a Schott filter, followed by washing with cold distilled water.

Synthesis of **3-Amino-5-(dimethylamino)-1-oxo-4,5,6,7,8,9-hexahydro-1*H*-pyrrolo[3,4-*c*]quinoline-3a,9b-dicarbonitrile (9a), 3-amino-5-(dimethylamino)-1-oxo-8-(propyl)-4,5,6,7,8,9-hexahydro-1*H*-pyrrolo[3,4-*c*]quinoline-3a,9b-dicarbonitrile (9b), 3-amino-5- (2-(dimethylamino)ethyl-1-oxo-4,5,6,7,8,9-hexahydro-1*H*-pyrrolo[3,4-*c*]quinoline-3a,9b-dicarbonitrile (9′), 2-(dimethylamino)-4a-methyl-10-oxo-5,6,7,8-tetrahydro-1H-8a,4-(epimino)quinoline-3,4(4*aH*)-dicarbonitrile (12c), (E)-2-amino-6,7,8,9,10,11,12,13-octahydro-5*H*-cyclododeca [*b*] pyran-3,4-dicarbonitrile (8)**.

The compounds were synthesized following a standard procedure, yielding 84% (0.21 g), 75% (0.18 g), 48% (0.12 g), 64% (0.14 g) and 62% (0.18 g), respectively. Initially, 8 mmol of **TCEK** in 5 mL of ethyl acetate was added to 8 mmol of **MDH** or **MDEA**, along with a catalytic amount of sodium hydroxide in 5 mL of ethyl acetate. The reaction progress was monitored using TLC, with *N,N*-dimethyl-*N’*-methylene hydrazine serving as the reference sample in terms of syntheses with TCEKs **1a,b,c, 2**, **MDEA**, used as a reference sample in terms of synthesis with TCEK **1a**. Then, 0.5–5 h later, sand-colored crystals precipitated. The desired product was separated by filtration through a Schott filter, followed by washing with cold ethyl acetate. Subsequently, recrystallization was performed using isopropanol as the solvent.

Synthesis of **2-(dimethylamino)-4a-methyl-10-oxo-5,6,7,8-tetrahydro-1*H*-8a,4-(epimino)quinoline-3,4(4*aH*)-dicarbonitrile (12c), (*E*)-2-amino-6,7,8,9,10,11,12,13-octahydro-5*H*-cyclododeca [*b*] pyran-3,4-dicarbonitrile (8)** without **MDH** catalysis.

Firstly, 8 mmol of **TCEK 2** was dissolved in 5 mL of ethyl acetate and allowed to react at room temperature for 24 h. Subsequently, the desired product precipitated out. The isolation and purification procedures were carried out following the same protocol as described above. The yield of the desired product was 54% (0.16 g).

In the specified quantities, the liberation of hydrogen cyanide does not present any danger. However, when dealing with larger quantities, it is imperative to utilize the aforementioned apparatus.

Synthesis of **5-Amino-1-(dimethylamino)-1,2-dihydro-3H-pyrrole-3,3,4-tricarbonitrile (12)**.

Firstly, 1.1 mmol of **MDH** was added to a solution of 1 mmol of **TCNEH_2_** in 2 mL of ethyl acetate. The mixture was kept at room temperature for 14 h (TLC control) and cooled. Precipitate was filtered off and washed with 2 mL of cold ethyl acetate. Yield: 81%.

## 5. Conclusions

Thus, 1,1-dimethylhydrazine can engage in reactions with various structural elements:

– Double bond (addition to the methylene-active position of tetracyanoketones via a Michael-type reaction);
– Dimethylamine fragment (rearrangement in the 2-methylcyclohexanone derivative);
– Labile hydrogen (addition to the double bond of tetracyanoethylene);
– Serving as a catalyst (accelerating the cyclization of cyclohexanone into a pyran derivative).

This makes it an indispensable starting compound for the synthesis of various organic structures. It should be noted that the exceptional availability of this reagent is of great significance.

## Figures and Tables

**Table 1:** Yield, melting point, IR, MNR ^1^H, ^13^C and mass-spectra data.

Structural Number	Yield, %	Melting Point, °C	IR (Nujol), ν_max_/cm^−1^	MNR^1^Hδ, ppm, J, Hz (DMSO-*d_6_*)	MNR ^13^C ppm, (DMSO-*d_6_*)	HRMS(ESI) and Mass-Spectra, *m*/*z*
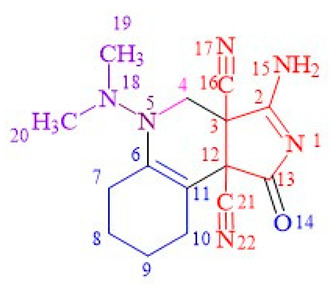 **9a**	84	217–219	1659 (s) (C=C), 1736 (vs.) (C=O), 2241 (w) (C≡N), 3291 (vs., br) (NH_2_)	9.63 (d, J = 30.32 Hz, 2H, NH_2_), 3.73 (d, J =11.67 Hz, 1H, CH_2_^4^), 3.34 (d, J = 11.75 Hz, 1H, CH_2_^4^), 2.33 (s, 6H, N(CH_3_)_2_), 2.28 (m, 2H, CH_2_^10^), 2.24–2.10 (m, 2H, CH_2_^7^), 1.65–1.54 (m, 2H, CH_2_^9^), 1.53–1.29 (m, 2H, CH_2_^8^).	178.15 (C=O), 177.07 (C^2^), 144.94 (C^6^), 116.16 (C≡N^21^), 115.24 (C≡N^16^), 95.07 (C^11^), 59.76 (C^3^), 52.94 (C^12^), 51.08 (CH_2_^4^), 39.52 (N(CH_3_)_2_), 24.92 (CH_2_^7^), 24.55(CH_2_^8^), 22.01 (CH_2_^9^), 21.88 (CH_2_^10^).	[M + H]^+^ 299.1620 (calculated for [C_15_H_19_N_6_O]^+^—299.1620).
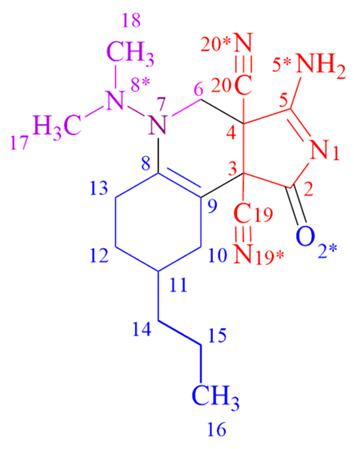 **9b**	75	214–216	1640 (s) (C=C), 1745 (s) (C=O), 2251 (s) (C≡N), 3494 (vs., br) (NH_2_)	9.61 (d, J = 15.42 Hz, 2H, NH_2_), 3.96 (d, J =11.66 Hz, 1H, CH_2_^6^), 3.20 (d, J = 11.63 Hz, 1H, CH_2_^6^), 2.47 (m, 2H, CH_2_^10^), 1.91 (t, J = 12.45 Hz, 2H, CH_2_^13^), 1.72 (m, 2H, CH_2_^12^), 1.49–1.42 (m, 2H, CH^11^), 1.38–1.28 (m, 2H, CH_2_^14^), 1.28–1.17 (m, 2H, CH_2_^15^), 1.04 (s, 3H, NCH_3_^17^), 1.03 (s, 3H, NCH_3_^18^), 0.88 (t, J = 7.01 Hz, 3H, CH_3_).	178.07 (C=O), 177.15 (C^5^), 145.03 (C^9^), 116.19 (C≡N^20^), 115.30 (C≡N^19^), 94.70 (C^8^), 62.07 (C^4^), 53.33 (C^3^), 51.57 (CH_2_^6^), 39.55 (N(CH_3_)_2_^17,18^), 37.85 (CH^6^), 31.91(CH_2_^14^, CH^11^), 28.41(CH_2_^13^), 25.33 ((CH_2_^10,12^)_2_), 19.63 (CH_2_^15^), 14.18 (CH_3_).	[M + H]^+^ 341.2090 (calculated for [C_18_H_24_N_6_O]^+^—341.2090).
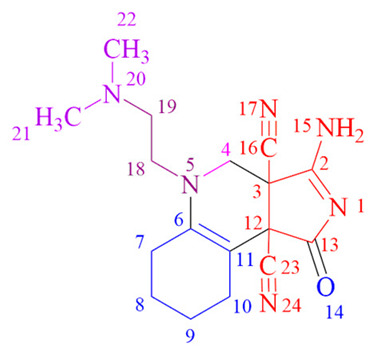 **9′**	62	235–236	1661 (s) (C=C), 1748 (vs.) (C=O), 2253 (w) (C≡N), 3291 (br), 3403 (w, br) (NH_2_)	9.60 (s, 2H, NH_2_), 3.66–3.48 (dd, 2H, J = 12.88, 7.66 CH_2_^4^), 3.25–3.00 (dh, 2H, J = 7.94, 1.88, CH_2_^18^), 2.29 (dh, J = 6.60, 1.53 Hz, 2H, CH_2_^19^), 2.18–2.15 (m, 4H, (CH_2_^7,10^), 2 2.14 (s, 6H, N(CH_3_)^2^), 1.54 (m, 2H, CH_2_^8^), 1.50 (m, 2H, CH_2_^9^).	179.31 (C=O), 177.80 (C^2^), 142.47 ((C^11,6^)_2_), 117.16 (C≡N^23^), 116.05 (C≡N^16^), 58.90 (CH_2_^19^), 54.11 (C^3^), 51.78 (C^12^), 51.11 (C^4^), 47.74 (CH_2_^18^), 46.24 (N(CH_3_)_2_), 25.94 (CH_2_^7^), 25.78 (CH_2_^8^), 22.93 (CH_2_^9^), 22.70 (CH_2_^10^).	Gas-chromatography mass-spectrum: [M+H]^+^ 326 (calculated for [C_17_H_22_N_6_O]^+^—326.1933)
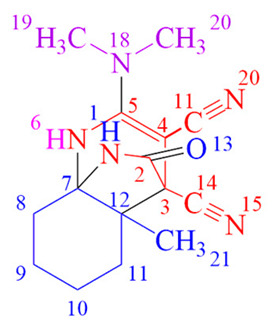 **12c**	48	>250	1579 (s) (C=C), 1717 (vs) (C=O), 2168 (vs) (=C-C≡N), 2242 (w) (C≡N), 3167 (br), 3282 (w) (NH)	8.42 (s, 1H, NH^1^), 7.06 (s, 1H, NH^6^), 2.91 (s, 6H, N(CH_3_)_2_), 2.03–1.07 (m, 8H, (CH_2_^12−7^)_4_), 0.89 (s, 3H, CH_3_)	168.68 (C=O), 157.75 (C^5^), 120.99 (C≡N^14^), 115.88 (C≡N^11^), 71.98 (C^5^), 54.29 (C^7^), 51.62 (C^12^), 40.67 (C^3^), 40.09 (N(CH_3_)_2_^19,20^), 31.86 (CH_2_^8^), 26.66 (CH_2_^11^), 20.86 (CH_2_^9^), 20.06 (CH_2_^10^), 13.72 (CH_3_).	[M + H]^+^ 286.1668 (calculated for [C_15_H_20_N_5_O]^+^—286.1668)
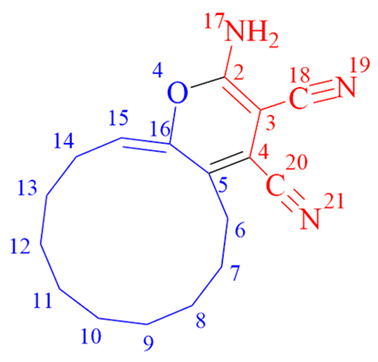 **8**	64	210–212	1547 (s) (C=C), 1658 (vs.) (C=C), 1731 (w) (=C-O), 2182 (s), 2233 (s) (C≡N), 3379 (w, br) (NH_2_)	8.12 (s, 2H, NH_2_), 5.27 (t, J = 8.16 Hz, 1H, CH^15^), 2.20 (q, J = 6.15 Hz, 2H, CH_2_^14^), 1.67–1.08 (m, 16H, (CH_2_^13−6^)_8_).	163.23 (C^2^), 145.43 (C^16^), 130.86 (C^5^), 116.93 (C^4^), 115.23 (CH^15^), 114.59 (C≡N^20^), 113.99 (C≡N^18^), 104.30 (C^3^), 28.33 (CH_2_^6^), 27.93 (CH_2_^7^), 27.53 (CH_2_^8^), 26.72 (CH_2_^9^), 26.43 (CH_2_^10^), 26.20 (CH_2_^11^), 25.85 (CH_2_^12^), 25.66 (CH_2_^13^), 25.09 (CH_2_^14^).	[M + H]^+^ 284.1763 (calculated for [C_17_H_22_N_3_O]^+^—284.1763)
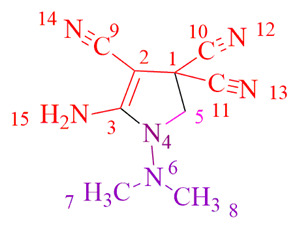 **12**	81	163–165	1646 (vs.) (C=C), 2112 (vs.), 2165 (s) (C≡N), 3177 (w, br), 3240 (w, br), 3362 (w, br), 3386 (w, br) (NH_2_)	7.56 (s, 2H, NH_2_), 4.05 (s, 2H, CH_2_^5^), 2.43 (s, 6H, N(CH_3_)_2_).	161.85 (C^3^), 117.70 (C≡N^9^), 115.14 ((C≡N^11,10^)_2_), 48.42 (CH_2_^5^), 46.42 (C^2^), 42.24 (N(CH_3_)^2^), 34.59 (C^1^)	[M + H]^+^ 203.1047 (calculated for [C_9_H_10_N_6_]^+^—203.1045)

**Table 2:** Crystal data and structure refinement for **9a**, **9b**, **12c** and **8**.

Identification Code	9a	9b	12c	8
Empirical formula	C_15_H_18_N_6_O	C_22_H_32_N_6_O_3_	C_15_H_19_N_5_O	C_17_H_21_N_3_O
Formula weight	298.35	428.53	285.35	283.37
Temperature/K	104(7)	102.2(9)	100(1)	99.9(10)
Crystal system	triclinic	triclinic	triclinic	triclinic
Space group	P-1	P-1	P-1	P-1
a/Å	13.9756(12)	9.42370(10)	7.5867(2)	8.5574(5)
b/Å	15.0773(15)	11.97650(10)	9.1339(3)	9.7008(6)
c/Å	18.0923(13)	11.99720(10)	10.8470(4)	9.7930(4)
α/°	112.010(8)	111.2460(10)	76.621(3)	102.032(4)
β/°	93.942(7)	111.1820(10)	82.305(3)	103.106(4)
γ/°	112.380(9)	93.9690(10)	81.452(3)	101.505(5)
Volume/Å^3^	3167.3(5)	1145.11(2)	719.26(4)	747.97(7)
Z	8	2	2	2
ρ_calc_mg/mm^3^	1.251	1.243	1.318	1.258
μ/mm^−1^	0.683	0.690	0.703	0.632
F(000)	1264.0	460.0	304.0	304.0
Crystal size/mm^3^	0.18 × 0.08 × 0.02	0.208 × 0.191 × 0.123	0.1 × 0.05 × 0.04	0.15 × 0.1 × 0.04
2Θ range for data collection	5.436 to 156.656°	8.14 to 153.026°	8.424 to 152.906°	9.618 to 153.2°
Index ranges	−17 ≤ h ≤ 17, −18 ≤ k ≤ 18, −15 ≤ l ≤ 22	−10 ≤ h ≤ 11, −14 ≤ k ≤ 14, −15 ≤ l ≤ 14	−9 ≤ h ≤ 8, −11 ≤ k ≤ 11, −12 ≤ l ≤ 13	−10 ≤ h ≤ 10, −12 ≤ k ≤ 12, −11 ≤ l ≤ 12
Reflections collected	34,121	33,743	17,589	7141
Independent reflections	12,415 [R(int) = 0.1673]	4548 [R(int) = 0.0349]	2865 [R(int) = 0.0345]	2981 [R(int) = 0.0614]
Data/restraints/parameters	12,415/0/801	4548/45/302	2865/0/193	2981/0/190
Goodness-of-fit on F^2^	1.003	1.034	1.021	1.090
Final R indexes [I ≥ 2σ (I)]	R_1_ = 0.0845, wR_2_ = 0.1731	R_1_ = 0.0422, wR_2_ = 0.1082	R_1_ = 0.0399, wR_2_ = 0.0966	R_1_ = 0.0821, wR_2_ = 0.2250
CCDC numbers	2,152,262	2,152,263	2,152,264	2,152,265

**Table 3:** Substituents **R_1_, R_2_, R_3_** and original compounds **1′a,b** and **1a–c**.

	1–9a	1–9b	1–6c	2′–9′
R_1_	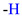	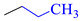	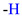	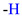
R_2_	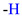	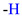	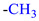	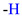
R_3_	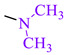	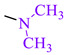	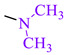	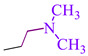
**1′**	**a**	**a**	**a**	**b**

## Data Availability

Not applicable.

## Supplementary Materials

The following supporting information can be downloaded at: https://www.mdpi.com/article/10.3390/ijms241713076/s1.
