# Supplementary material for: Treatment of Substandard Rocket Fuel 1,1-Dimethylhydrazine via Its Methylene Derivative into Heterocycles Based on Pyrrolo-[3,4c]Quinolines, Cyclododeca[b]piran and Pyrrole
Source: Int J Mol Sci. 2023 Aug 22;24(17):13076. doi: 10.3390/ijms241713076 (PMC10487424; doi:10.3390/ijms241713076)
Supplement: Supplementary file 1 [file ijms-24-13076-s001.zip › ijms-2502830-supplementary.pdf]

Supporting information

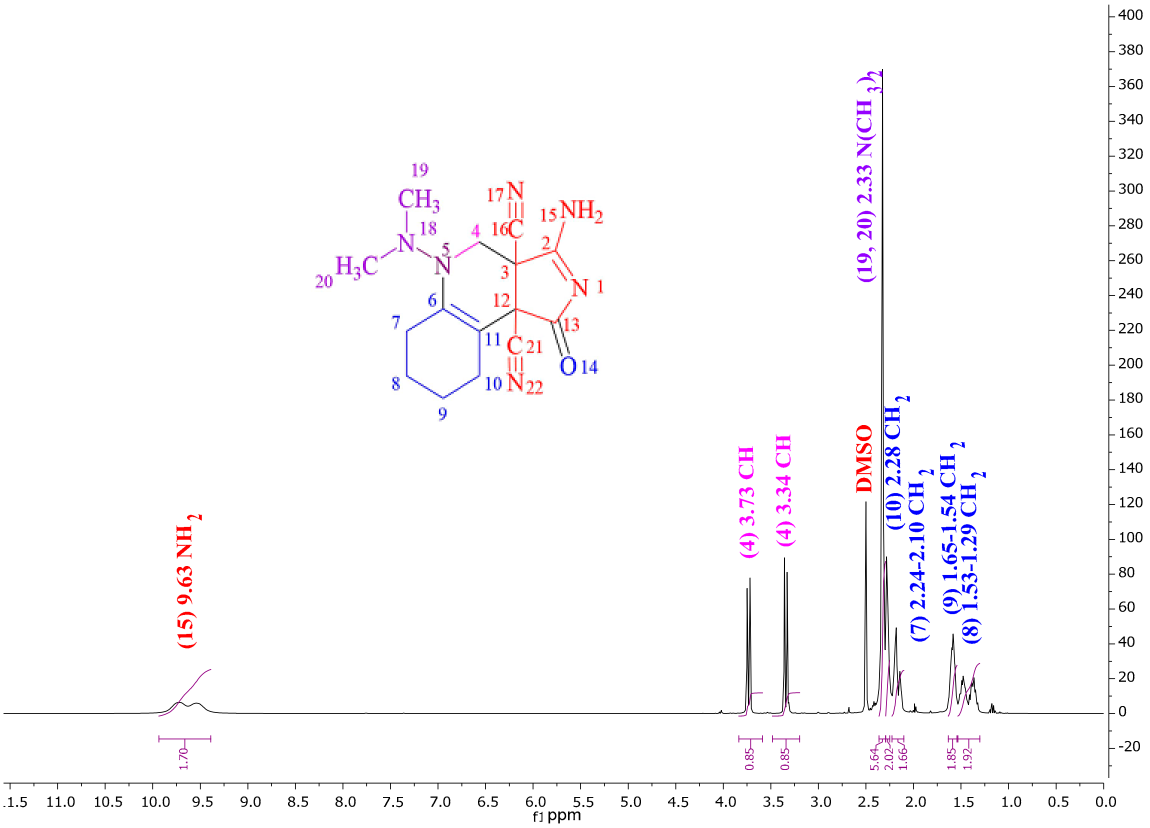

Figure S1. ^{1}H NMR-spectrum of **9a** (400 MHz, DMSO-d6, 298K)

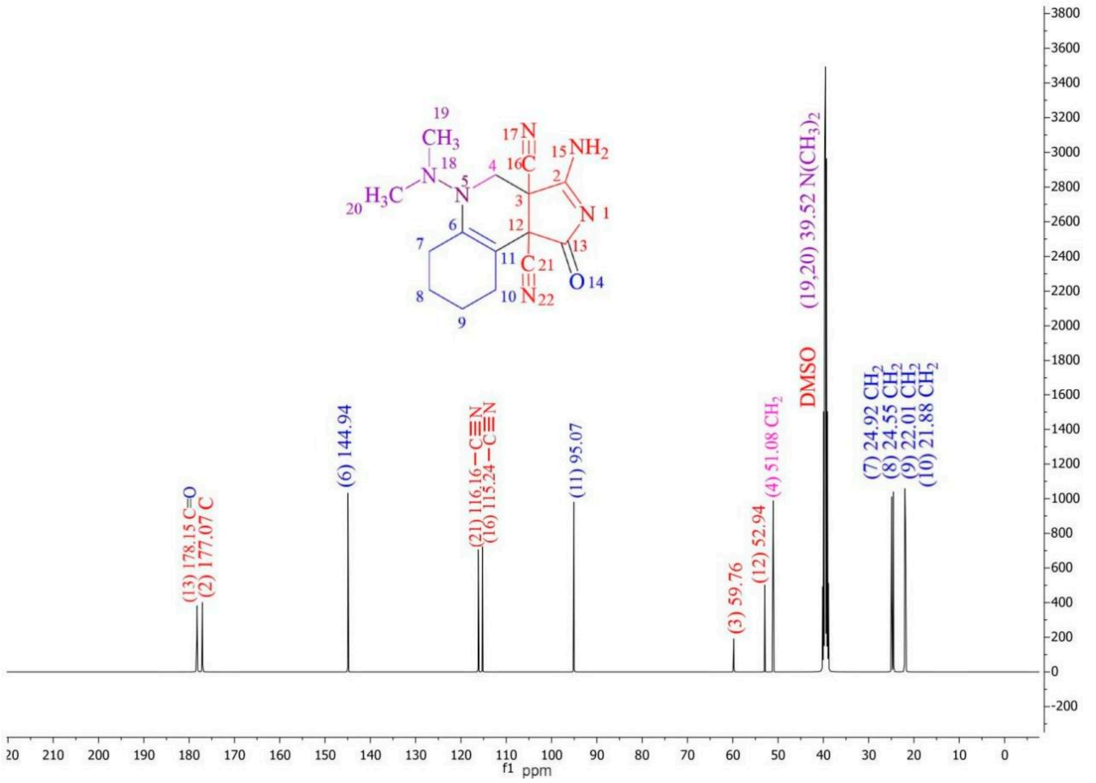

Figure S2. ^{13}C NMR-spectrum of **9a** (100 MHz, DMSO-d6, 299K)

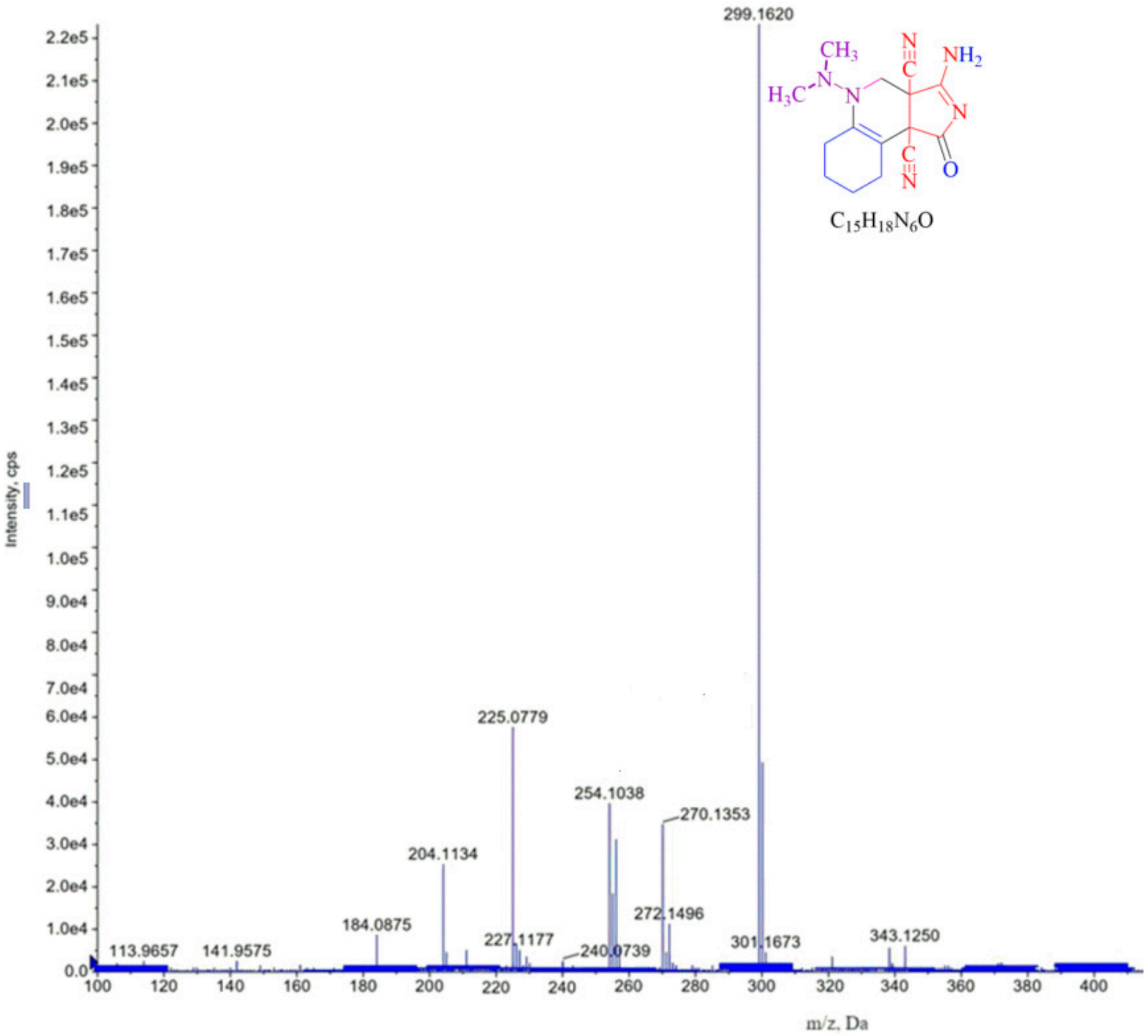

Figure S3. HRMS-spectrum of **9a**

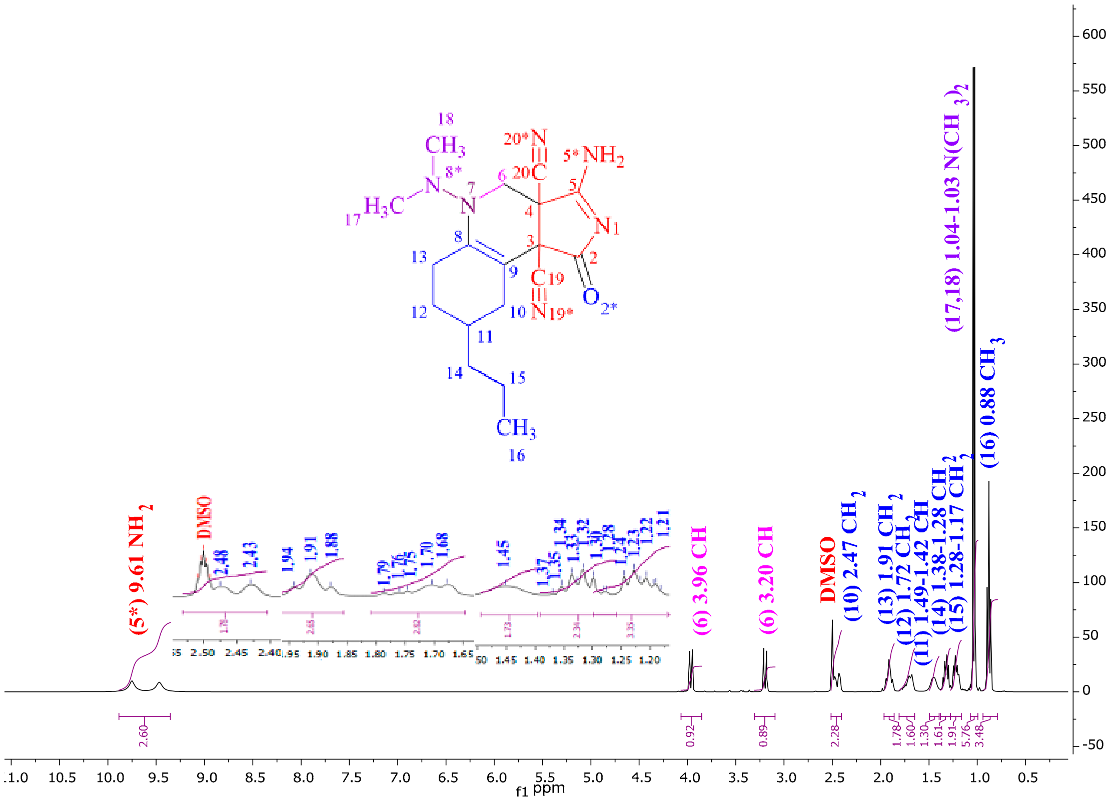

Figure S4. ^{1}H NMR-spectrum of **9b** (400 MHz, DMSO-d6, 299K)

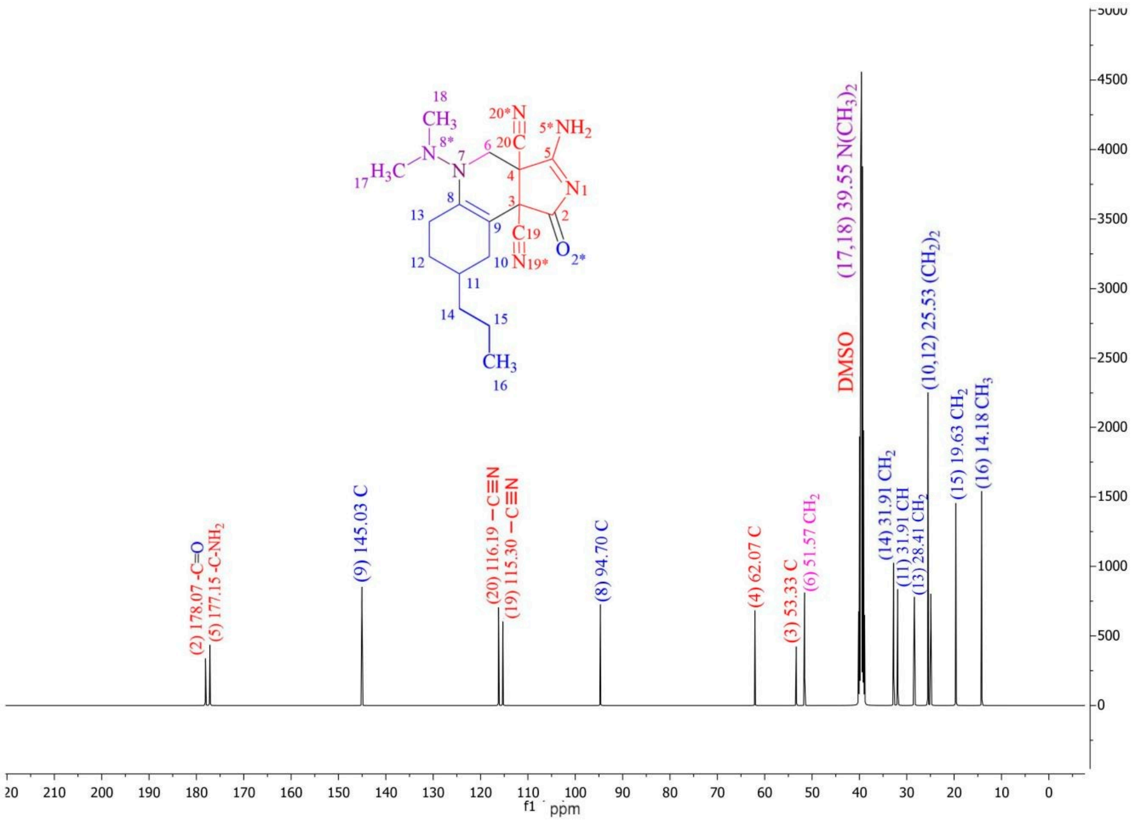

Figure S5. ^{13}C NMR-spectrum of **9b** (100 MHz, DMSO-d6, 299K)

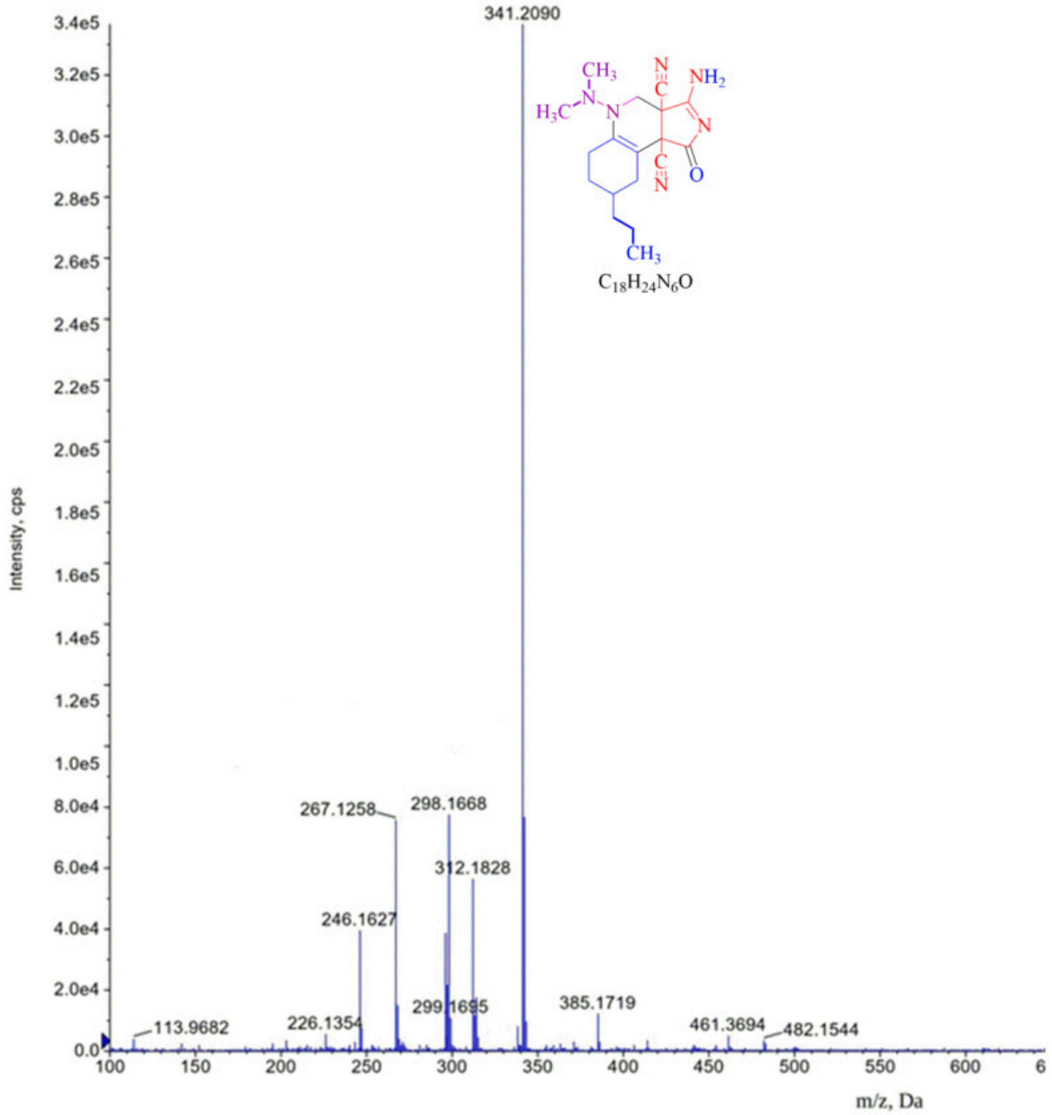

Figure S6. HRMS-spectrum of **9b**

f

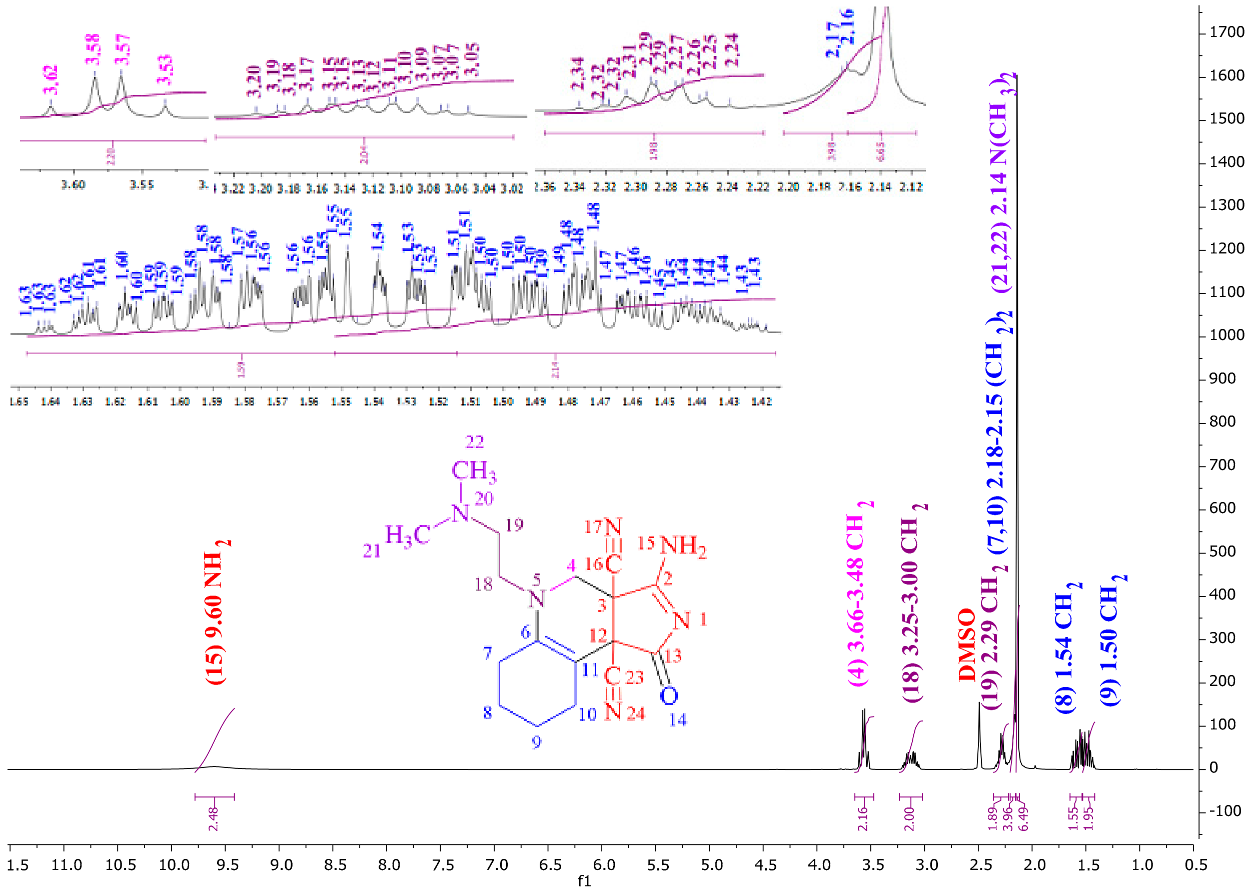

Figure S7. ^{1}H NMR-spectrum of **9`** (400 MHz, DMSO−d6, 297K)

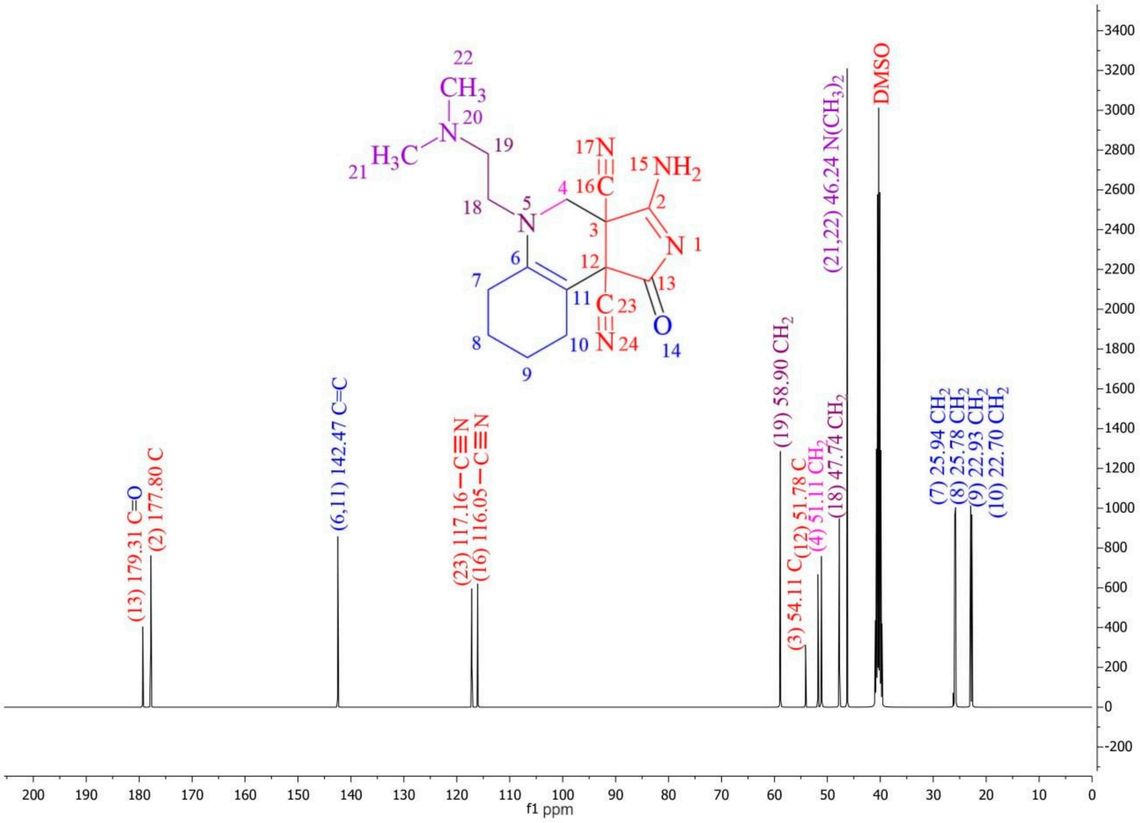

Figure S8. ^{13}C NMR-spectrum of **9`** (100 MHz, DMSO-d6, 297K)

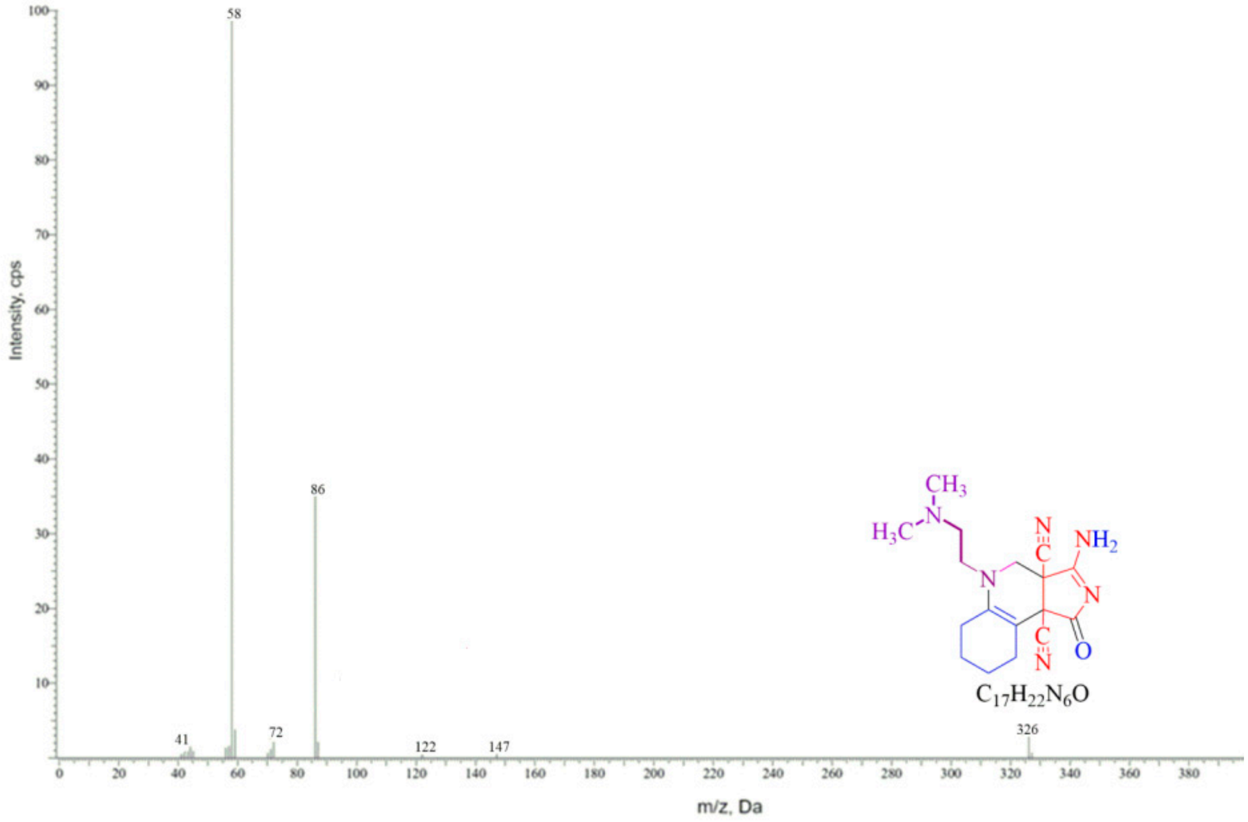

Figure S9. Mass spectra of **9`**

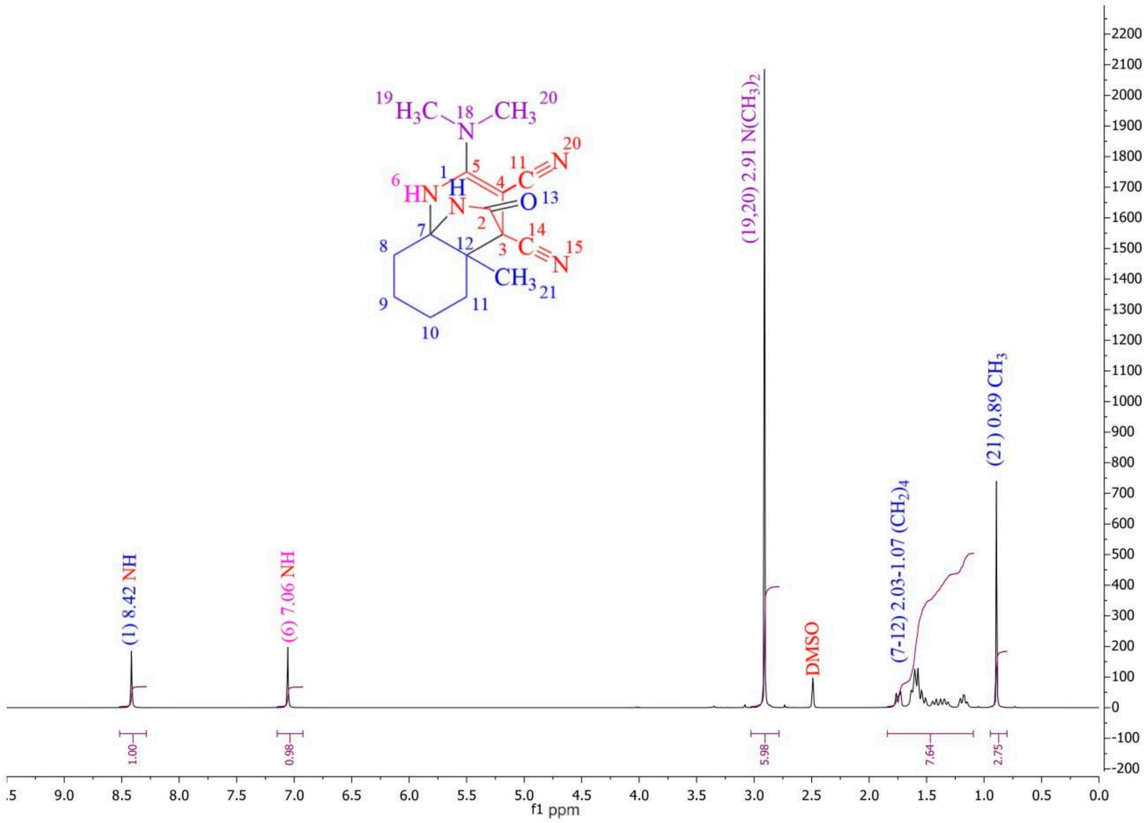

Figure S10. ^{1}H NMR-spectrum of **12c** (400 MHz, DMSO-d6, 296K)

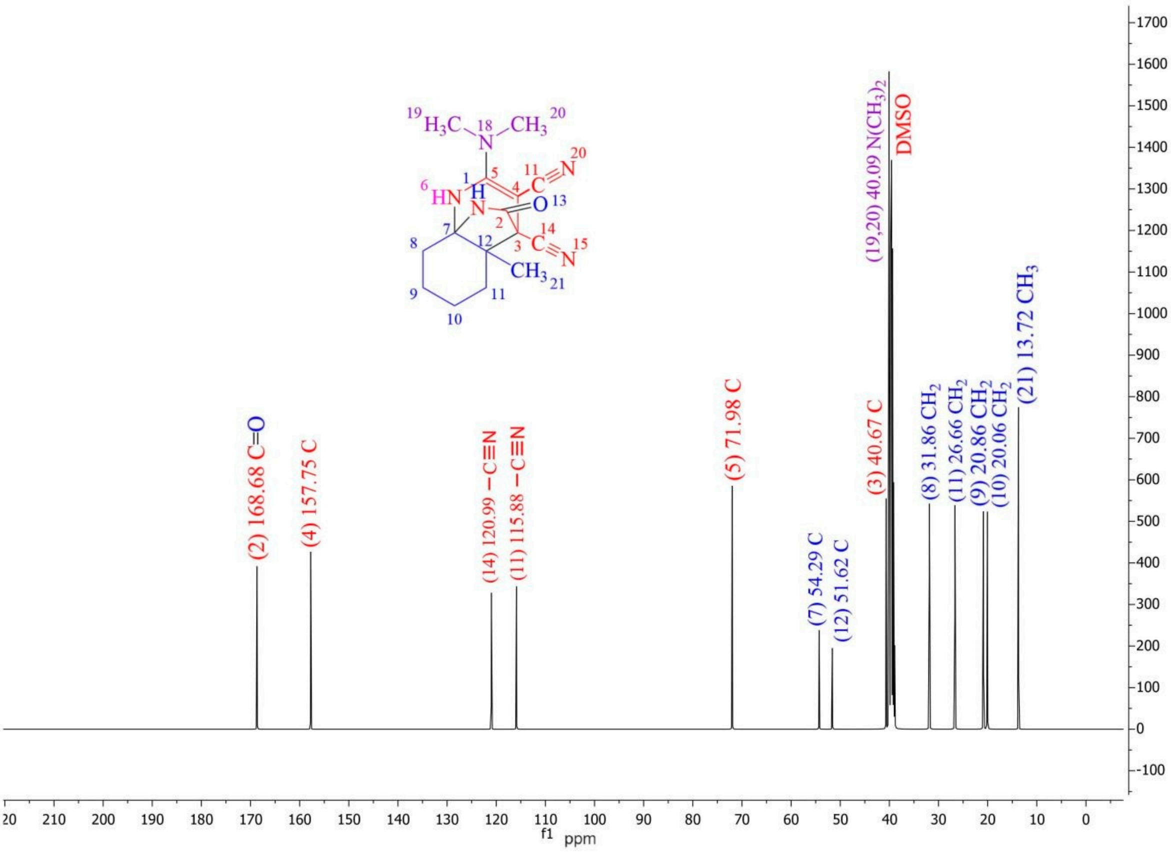

Figure S11. ^{13}C NMR-spectrum of **12c** (100 MHz, DMSO-d6, 297K)

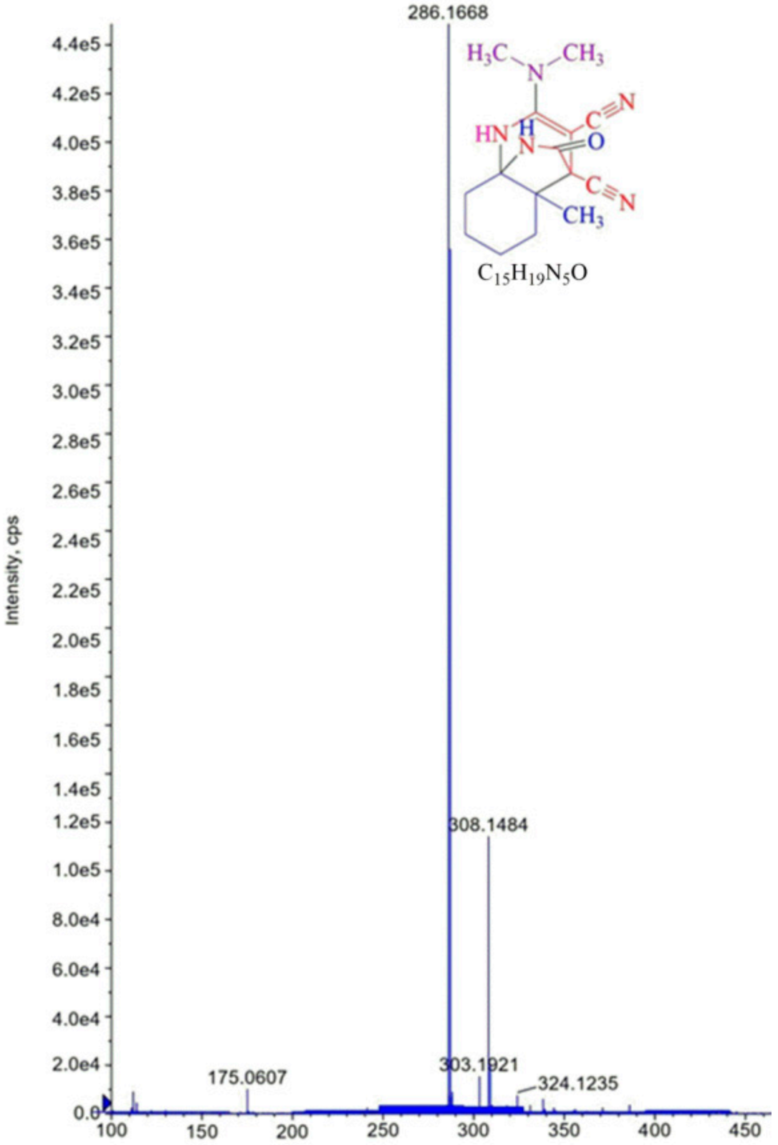

Figure S12. HRMS-spectrum of **12c**

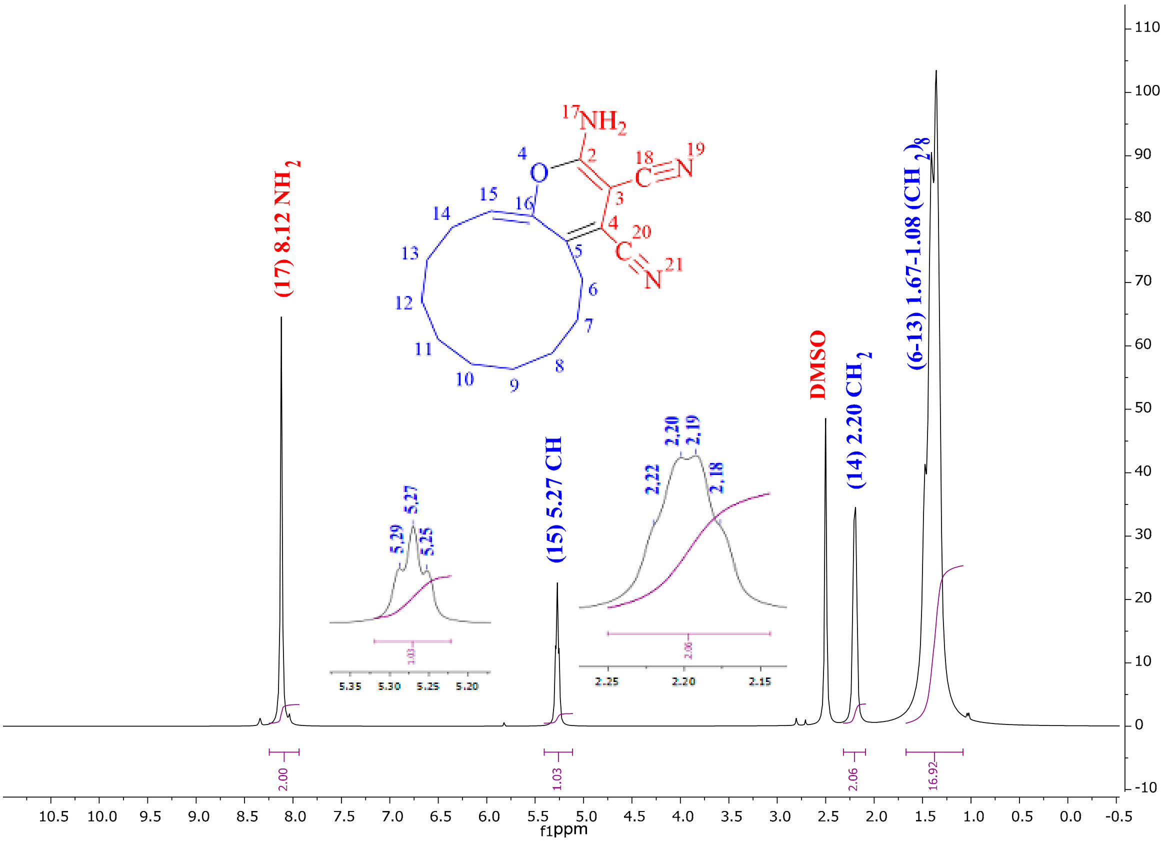

Figure S13. ^{1}H NMR-spectrum of **8** (400 MHz, DMSO-d6, 296K)

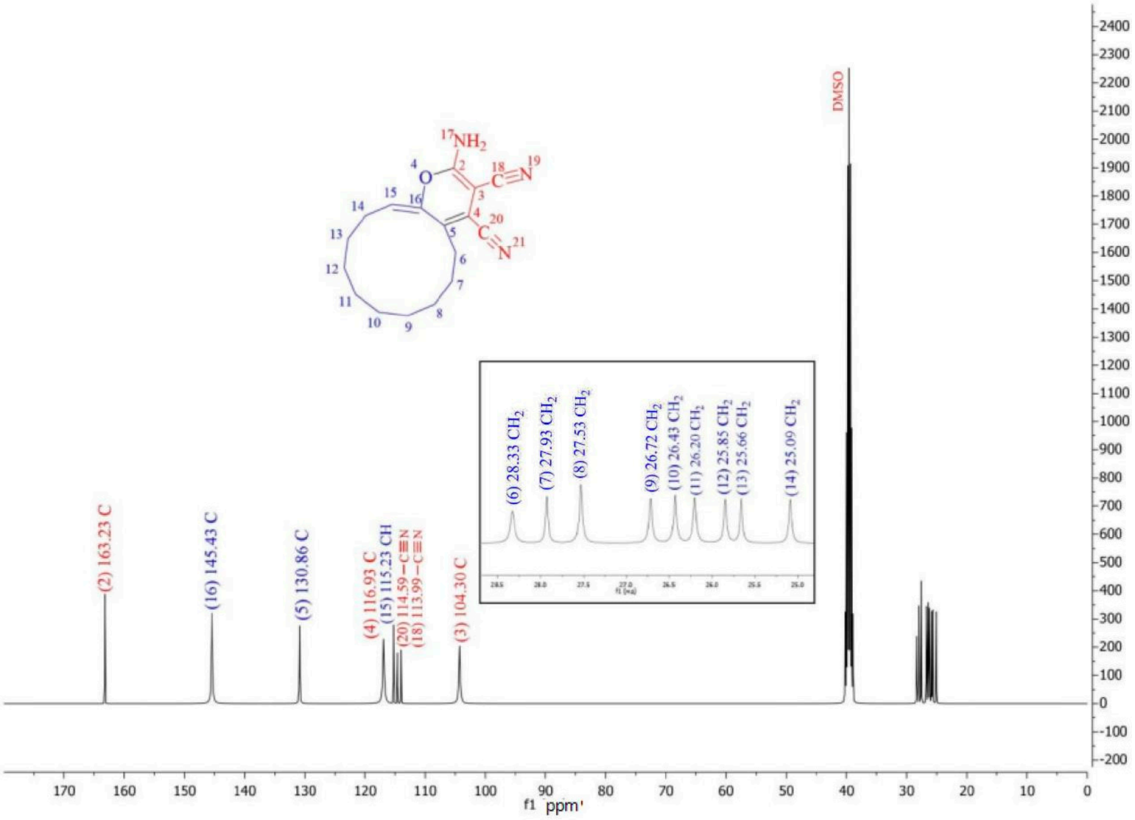

Figure S14. ^{13}C NMR-spectrum of **8** (100 MHz, DMSO-d6, 297K)

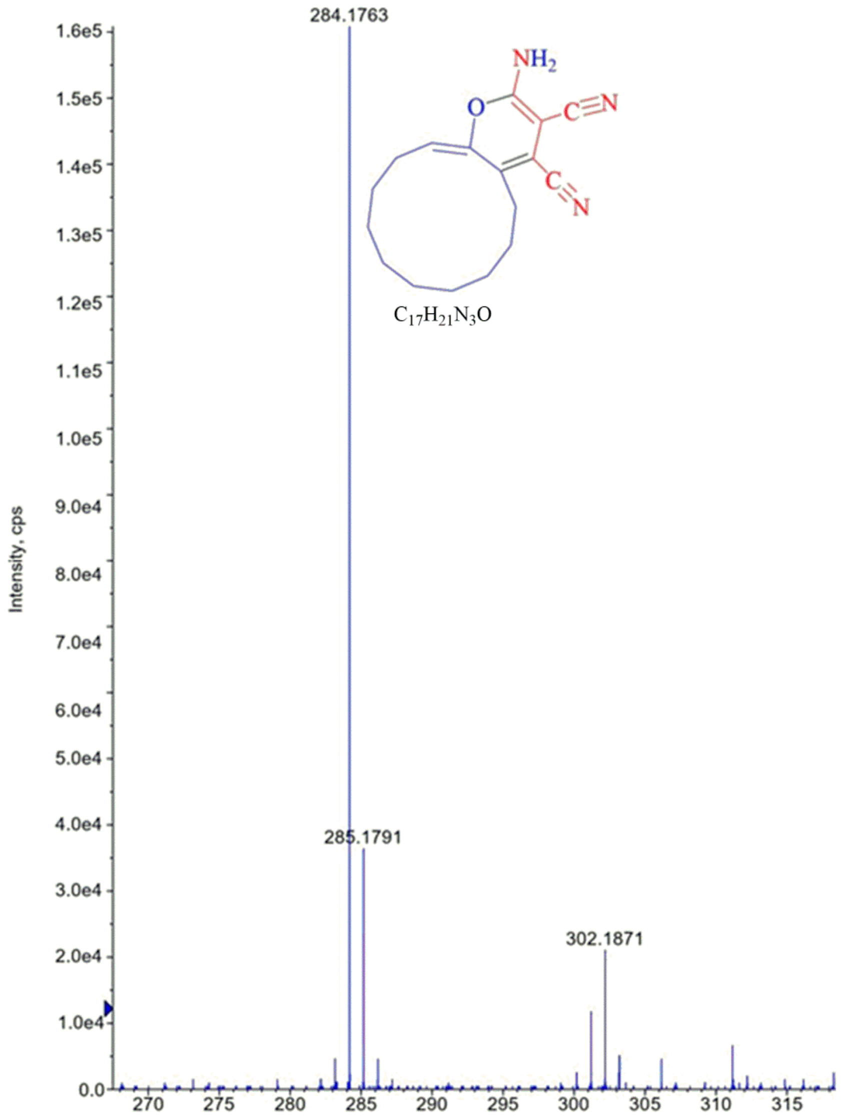

Figure S15. HRMS of **8**

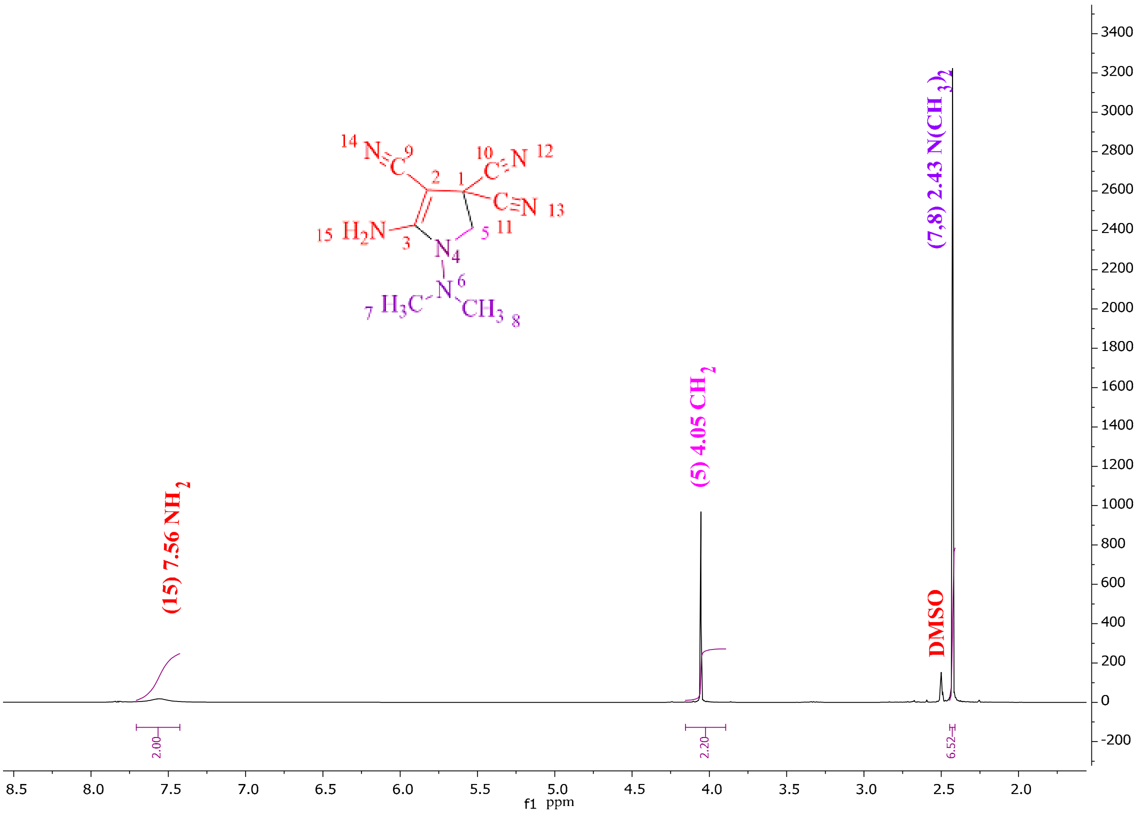

Figure S16. ^{1}H NMR-spectrum of **12** (400 MHz, DMSO-d6, 297K)

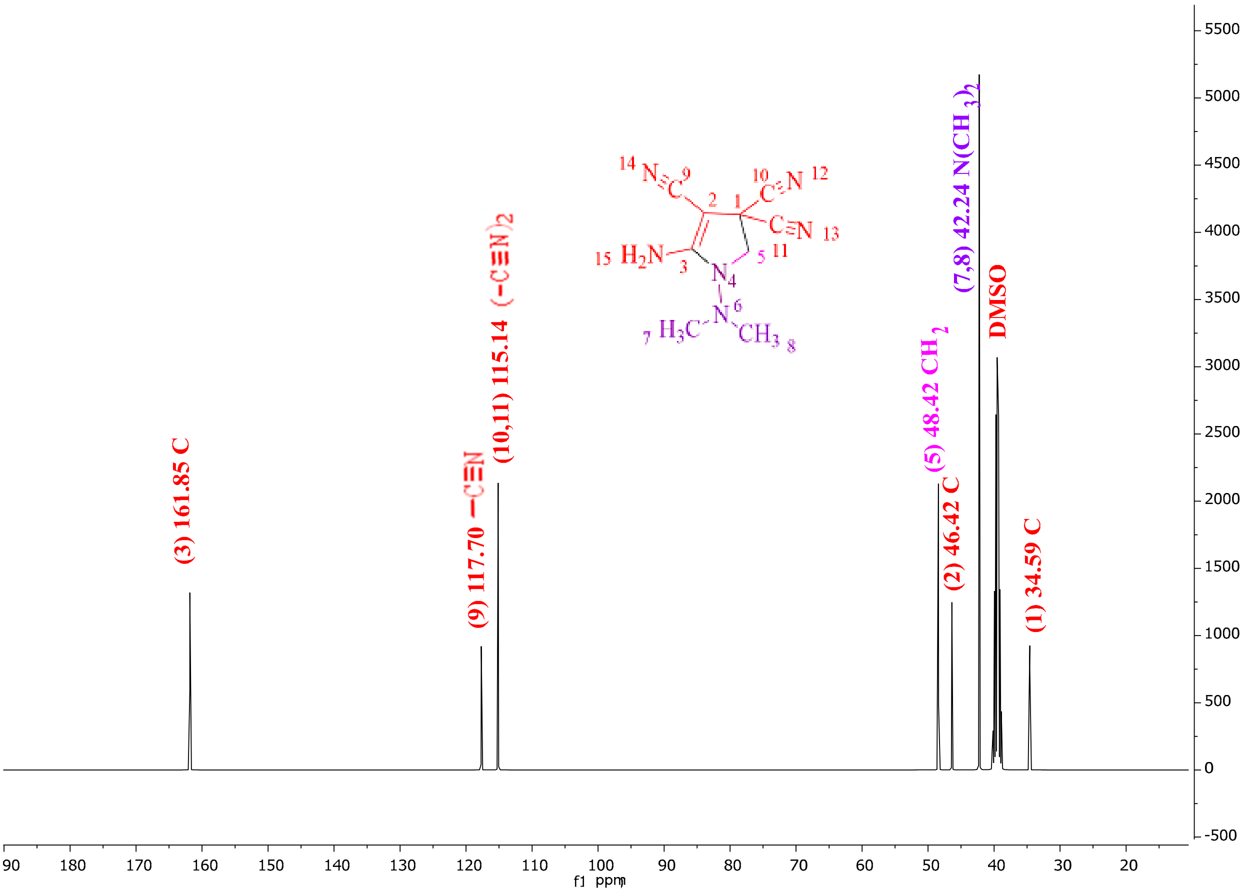

Figure S17. ^{13}C NMR-spectrum of **12** (100 MHz, DMSO-d6, 297K)

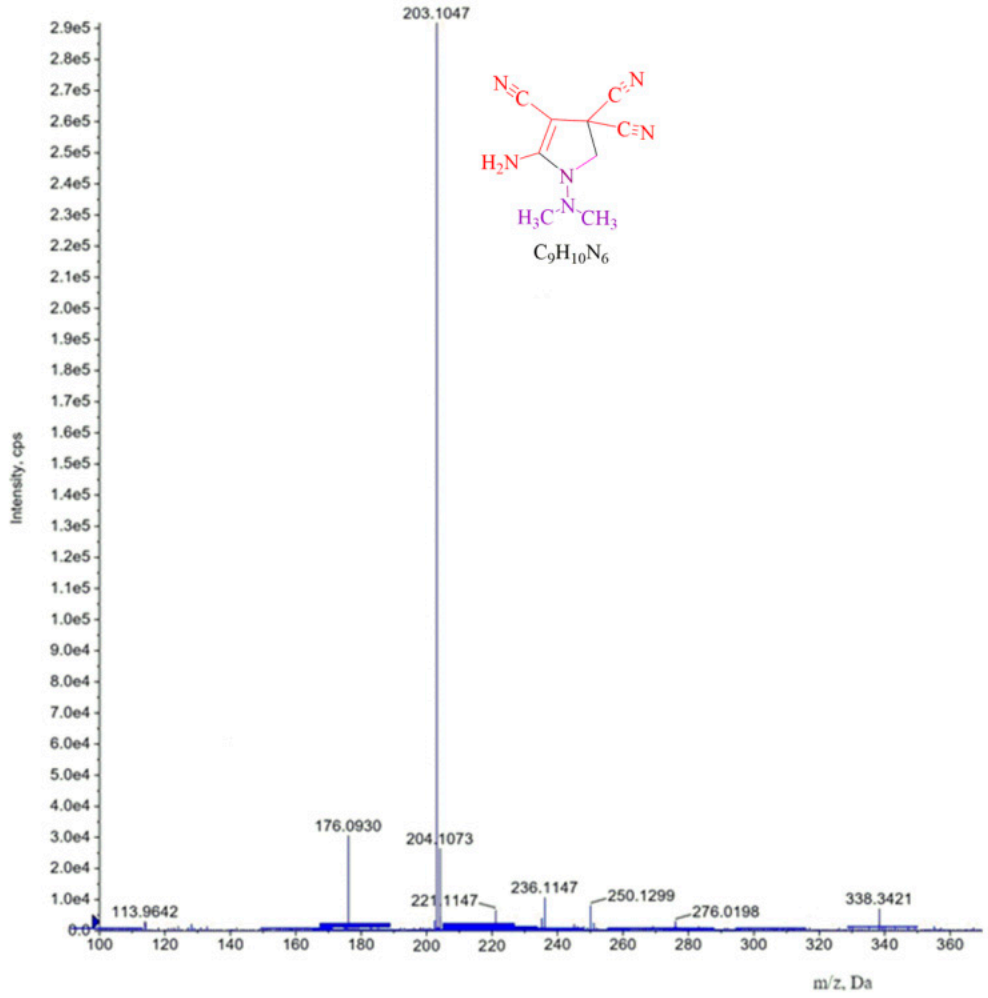

Figure S18. HRMS of **12.**
